# Biomarkers in Systemic Juvenile Idiopathic Arthritis, Macrophage Activation Syndrome and Their Importance in COVID Era

**DOI:** 10.3390/ijms232112757

**Published:** 2022-10-22

**Authors:** Laura Marinela Ailioaie, Constantin Ailioaie, Gerhard Litscher

**Affiliations:** 1 Department of Medical Physics, Alexandru Ioan Cuza University, 11 Carol I Boulevard, 700506 Iasi, Romania; 2 Research Unit of Biomedical Engineering in Anesthesia and Intensive Care Medicine, Research Unit for Complementary and Integrative Laser Medicine, Traditional Chinese Medicine (TCM) Research Center Graz, Department of Anesthesiology and Intensive Care Medicine, Medical University of Graz, Auenbruggerplatz 39, 8036 Graz, Austria

**Keywords:** chemokines, cytokines, cytokine storm syndrome, ferritin, hub genes, innate immunity, natural killer, neutrophils, platelets, S100 proteins, biomarkers, systemic juvenile idiopathic arthritis, COVID-19

## Abstract

Systemic juvenile idiopathic arthritis (sJIA) and its complication, macrophage activation syndrome (sJIA-MAS), are rare but sometimes very serious or even critical diseases of childhood that can occasionally be characterized by nonspecific clinical signs and symptoms at onset—such as non-remitting high fever, headache, rash, or arthralgia—and are biologically accompanied by an increase in acute-phase reactants. For a correct positive diagnosis, it is necessary to rule out bacterial or viral infections, neoplasia, and other immune-mediated inflammatory diseases. Delays in diagnosis will result in late initiation of targeted therapy. A set of biomarkers is useful to distinguish sJIA or sJIA-MAS from similar clinical entities, especially when arthritis is absent. Biomarkers should be accessible to many patients, with convenient production and acquisition prices for pediatric medical laboratories, as well as being easy to determine, having high sensitivity and specificity, and correlating with pathophysiological disease pathways. The aim of this review was to identify the newest and most powerful biomarkers and their synergistic interaction for easy and accurate recognition of sJIA and sJIA-MAS, so as to immediately guide clinicians in correct diagnosis and in predicting disease outcomes, the response to treatment, and the risk of relapses. Biomarkers constitute an exciting field of research, especially due to the heterogeneous nature of cytokine storm syndromes (CSSs) in the COVID era. They must be selected with utmost care—a fact supported by the increasingly improved genetic and pathophysiological comprehension of sJIA, but also of CSS—so that new classification systems may soon be developed to define homogeneous groups of patients, although each with a distinct disease.

## 1. Introduction

Juvenile idiopathic arthritis (JIA) is a set of chronic childhood disorders that usually cause inflammation and pain, swelling, and stiffness in the joints of children under the age of 16 years, which may last from months to the entire life of the patient, with a risk of locomotor and ocular disabilities. The unparalleled therapeutic advances since the beginning of this century have turned the remission or minimization of disease activity from an ideal goal to an achievable one for most JIA patients, with fewer joint and extra-articular injuries and reduced incidence of long-term physical disabilities. However, with all current therapeutic means of conventional synthetic disease-modifying anti-rheumatic drugs (csDMARDs) and/or biological DMARDs (bDMARDs), long-term remission is difficult to achieve for some patients [1,2,3].

Nevertheless, there are scientific reports indicating that after 5 years from diagnosis, less than half of JIA patients who received contemporary medication went into remission. At the same time, prolonged anti-inflammatory treatment is associated with high financial costs, high stress for the patient and their family, and potential side effects [4,5].

Current diagnostic guidelines for patients with JIA are not currently accompanied by recommendations for the use of predictive molecular biomarkers for relapses, complications, disease progression, and prognosis.

Action plans based on predictive biomarkers are promising tools in decision-making as to whether to withdraw or discontinue medication if the inflammation is no longer severe enough to show clear clinical symptoms, and could be very beneficial to patients [6].

Systemic juvenile idiopathic arthritis (sJIA) is a subtype manifested by arthritis or arthralgia, in addition to extra-articular symptoms, which include the following: high daily and prolonged fever of unknown origin, persisting for at least 3 consecutive days and recurring for at least 2 weeks; transient rash; and at least one of the following clinical characteristics: enlarged lymph nodes; hepatosplenomegaly; or pleural, pericardial, or peritoneal effusion, sometimes associated with pneumonitis and/or signs of central nervous system damage. sJIA, previously thought to be an autoimmune disease, is now more commonly classified as an autoinflammatory disease, because the genetic abnormalities of the major histocompatibility complex (MHC) and the innate immune system (such as natural killer (NK) cells, polymorphonuclear neutrophils (PMNs), and macrophages (MP))—with the participation of interleukins 1 (IL-1), 6 (IL-6), and 18 (IL-18)—have been shown to be involved. The prognosis and evolution of the disease are unpredictable, ranging from a mild single-phase evolution to a severe chronic cyclic polyarticular disease complicated by extra-articular manifestations that can induce significant morbidity and mortality [7,8,9,10,11].

sJIA has many characteristics of an autoinflammatory pathology, associated with an increased synthesis of pro-inflammatory interleukins such as IL-1, IL-6, and IL-18, as well as S100 proteins; at the same time, in its evolution, sJIA can be complicated by macrophage activation syndrome (MAS). This term is used by rheumatologists to describe the aggressive, life-threatening complications in patients with sJIA or AOSD, and is phenotypically associated with secondary hemophagocytic lymphohistiocytosis (sec-HLH), in which IFN-γ and CXCL9 play essential roles as biomarkers. MAS is manifested by high fever, cytopenia, cytokine storm (CS), severe liver dysfunction, consumptive coagulopathy, seizures, coma, and even death. During the evolution of sJIA as active disease, MAS may occur (sJIA-MAS) in 7–17% of cases, while subclinical cases are even more frequent, found in 30–40% of patients. The criteria for the classification of sJIA-MAS were advanced in 2016 [12,13,14,15,16].

Biomarkers have a high potential as indispensable tools for pediatric rheumatologists in the early differentiation of cytokine storm syndromes (CSSs).

Currently, due to the diversity of cases and the increasingly complicated pathologies that appear after infection with SARS-CoV-2—such as multisystem inflammatory syndrome in children (MIS-C)—the need to refine and maximize these biomarkers has become more and more important, in order to move from the laboratory to the patient’s bedside as soon as possible, to apply the results of research in as many clinical laboratories as possible for sampling the analyses. Based on this approach, a successful positive diagnosis and the early initiation of the appropriate individualized treatment for the respective patient could save their life in the event of CSS-aggravating circumstances.

The aim of this review was to identify the newest and most powerful biomarkers and their synergistic interactions for easy and accurate recognition of sJIA, as well as its complication sJIA-MAS, to immediately guide clinicians in the correct diagnosis and in predicting the outcomes of the disease, the response to treatment, and the risk of relapses.

Biomarkers constitute an exciting field of research, especially due to the heterogeneous nature of CSS in the COVID-19 era. They must be selected with the utmost care—a fact supported by the increasingly improved genetic and pathophysiological comprehension of sJIA, but also of CSS—so that new classification systems can be developed to define homogeneous groups of patients, each with a distinct disease.

The discovery of new biomarkers with the best possible diagnostic/prognostic value could increase the speed of reaching the final goal, i.e., the introduction of new targeted therapies and personalized interventions, improving disease remission percentages while minimizing all undesirable pathophysiological effects of the disease.

## 2. Biomarkers for Diagnosis, Treatment, and Disease Activity in sJIA Complicated by MAS

A positive diagnosis of sJIA is initially very difficult, especially when arthritis is absent as a defining symptom. In this case, it is necessary to extend the investigations by making a differential diagnosis with different entities that are caused by viral or bacterial infections, neoplasms, and other inflammatory diseases. It is precisely these issues that generate the urgent need to find biomarkers to help exclude the various other entities for the early diagnosis of sJIA. These biomarkers must have high sensitivity and specificity, be easy to determine at low cost, and be found in the pathogenic mechanisms of the disease.

The absence of arthritis at the beginning of the disease is an impediment for clinicians, as there are currently no reliable diagnostic criteria, and this could lead to a delay in early positive diagnosis, which may have consequences for the initiation of targeted treatment, as well as patients’ outcomes and prognosis [17,18].

The discovery of the roles of IL-1 and IL-6 in the pathogenesis of sJIA had the effect of introducing an extremely effective therapy—especially if administered at the onset of the disease—by targeting these cytokines. However, 20–30% of cases do not respond to initial therapy with anti-IL-1 or anti-IL-6 biological agents, leading to the urgent need to discover new biomarkers for diagnosis, develop a more effective management plan, and accurately predict the course and prognosis of the disease [7,19].

### 2.1. Genetic Biomarkers

Perception of the genetic hazard posed by sJIA—which, albeit less frequent than the other subtypes of JIA and with an unusual clinical expression at the first visit, can evolve in a severe manner that is sometimes complicated by MAS—has initiated important research to determine the genetic susceptibility to sJIA, as a challenge in a neglected area of investigation, so as to be able to more easily identify genetic biomarkers for early diagnosis, establishing a targeted treatment from the beginning and accurately predicting the long-term response to therapy for these patients.

For 136 sJIA patients at onset, De Benedetti et al. assessed the association of the MIF-173 polymorphism with patients’ long-term response, observed for more than 5 years. The levels of MIF-173 single-nucleotide G-to-C polymorphism of the macrophage migration inhibitory factor (MIF) gene in the serum and synovial fluid of patients with sJIA were studied in correlation with the required amount of glucocorticoids and the outcomes in sJIA patients. Subjects carrying the MIF-173*C allele had significantly higher serum and synovial concentrations of MIF than those with the GG genotype; therefore, the former needed a much longer treatment with glucocorticoids than in MIF-173 GG homozygous patients and had a shorter duration of clinical response to intraarticular injections. Finally, the number of joints with active arthritis, the questionnaires scores, and the number of joints with limited range of motion were significantly higher in patients carrying the MIF-173*C allele, so this could predict poor outcomes of sJIA at onset [20].

Starting from the fact that the polymorphisms in the interferon regulatory factor 5 (*IRF5*) gene have been linked with susceptibility to autoimmune diseases (ADs), Yanagimachi et al. intended to evaluate the associations of *IRF5* gene polymorphisms with the vulnerability to sJIA and MAS. Using TaqMan assays, the authors genotyped three *IRF5* single-nucleotide polymorphisms (rs729302, rs2004640, and rs2280714) in 81 patients with sJIA (33 with MAS, 48 without) and 190 controls. A significant association of the rs2004640 T allele with the vulnerability to MAS was highlighted. The *IRF5* haplotype (rs729302 A, rs2004640 T, and rs2280714 T), known to be an increased risk factor in systemic lupus erythematosus (SLE), was also significantly associated with susceptibility to MAS in sJIA. *IRF5* gene polymorphism influences susceptibility to MAS in sJIA, so *IRF5* could trigger MAS in sJIA [21].

At the level of the innate immune system, inflammasomes receive and respond to the signals of the pathological factors, as well to the injuries produced through the activation of caspase-1, the secretion of IL-1β and IL-18, and pyroptosis of macrophages; as a result, Canna et al. found a de novo missense mutation, c.1009A > T, p.Thr337Ser, in the nucleotide-binding domain of the NLRC4 inflammasome that causes early-onset relapsing fever and MAS. Operational analyses shed light on the spontaneous generation of the inflammasome, along with the production of IL-1β- and IL-18-dependent cytokines, revealing a new monoallelic inflammasome defect that broadens the field of monogenic ADs—including MAS—and opens the possibility of new targets in therapy [22].

Ombrello et al. investigated whether genetic variation in the MHC locus on chromosome 6 influenced the susceptibility to the onset of sJIA in a group of 982 children with sJIA and 8010 healthy control subjects from nine countries. The authors used a meta-analysis of directly observed and imputed single-nucleotide polymorphism (SNP) genotypes and imputed classical HLA types, finding that the strongest SNP associated with sJIA was rs151043342 from the HLA class II region. Meta-analysis of the imputed classical HLA-type associations showed that HLA-DRB1*11 (conferring at least a twofold increase in disease risk in each population studied) and its defining amino acid residue—glutamate 58—were strongly associated with sJIA, as were the HLA-DRB1*11 haplotypes, i.e., HLA-DQA1*05–HLA-DQB1*03. This research demonstrated the relationship between the HLA class II region and sJIA, as well as the participation of adaptive immune molecules in the etiopathogenesis of sJIA [23].

In the etiopathogenesis of JIA, the genetic predisposition mainly concerns HLA class II molecules (e.g., HLA-DRB1, HLA-DPB1), although HLA class I molecules and non-HLA genes have also been found to be involved. A meta-analysis of selected papers for the evaluation of the HLA-DRB1 genetic background of patients with JIA compared to healthy controls, conducted by De Silvestri et al., confirmed that HLA-DRB1*04 has a predisposing role in sJIA [24].

Arthur et al., with the largest cohort at the time, investigated whether susceptibility loci previously determined through studies on candidate genes in JIA had a provable association with sJIA. The authors studied SNPs for risk loci in sJIA by association in nine populations, which incorporated 770 patients with sJIA and 6947 controls. The results demonstrated that among the 26 previously reported SNPs there was only one notable association: the promoter region of IL1RN. The *IL1RN* gene encodes the interleukin-1 receptor antagonist (IL-1Ra). sJIA-associated SNPs are well-correlated with *IL1RN* expression in lymphoblastoid cell lines (LCLs), with an inverse correlation between sJIA risk and *IL1RN* expression. The high expression of homozygous *IL1RN* alleles is strongly correlated with the lack of response to anakinra therapy, paving the way for personalized treatments in sJIA guided by homozygous high-expression alleles with the attributes of ideal biomarkers [25].

Recent advances in “omics” technologies (e.g., genomics, transcriptomics, proteomics, and metabolomics) have made innovative contributions to human genome sequencing, bioinformatics, and analytical tools, significantly facilitating knowledge of the molecular mechanisms of JIA and providing the potential to discover new therapeutic agents. In a review on the multi-omics architecture of JIA, Hou et al. identified several human leukocyte antigen (HLA) alleles (including both HLA class I and class II genes) and 23 non-HLA genetic loci in association with different JIA subtypes. HLA class II alleles are remarkably associated with sJIA. HLA-DRB1*11 has a strong association, while the SNP rs151043342 has the strongest association with sJIA [26].

Zhou et al. attempted to identify key modules and hub genes in the pathophysiology of sJIA, using two datasets on GSE7753 and GSE13501, to accomplish a weighted gene co-expression network analysis (WGCNA). The authors identified a total of 5414 genes for WGCNA, of which the highly correlated ones were divided into 17 modules, where the red module had the highest correlation with sJIA, while the green–yellow one was not related to sJIA. The red module was enriched in the activation of immune responses, infection, nucleosomes, and erythrocyte differentiation, including the *ALAS2*, *AHSP*, *TRIM10*, *TRIM58*, and *KLF1* genes, while the green–yellow module was enriched in inflammation and immune responses, such as the *KLRB1*, *KLRF1*, *CD160*, and *KIR* genes. These results may deepen the comprehension of the pathophysiology of sJIA, and hub genes may play a role as biomarkers and future treatment targets for sJIA [27]. 

Due to the fact that sJIA is a severe disease, with an as-yet undetermined etiology and atypical manifestations at the onset, it requires diagnosis and effective treatment from the beginning. In this regard, Zhang et al. aimed to identify diagnostic biomarkers, immune cells, and pathways involved in the pathogenesis of sJIA, as well as possible treatment targets. The authors checked the gene expression profiles of GSE17590, GSE80060, and GSE112057 from the Gene Expression Omnibus (GEO) database to identify differentially expressed genes (DEGs) between sJIA and healthy controls; they analyzed whole-blood samples from 10 subjects, including 5 patients with sJIA (1 very recently diagnosed and 4 already undergoing treatment) and 5 healthy controls perfectly matched for age, race, and sex. The results identified 73 common DEGs, indicating the enrichment of neutrophil and platelet functions as well as the MAPK pathway in the pathogenesis of sJIA. Six hub genes were identified, three of which had high diagnostic sensitivity and specificity; *ARG1* and *PGLYRP1* were validated by quantitative reverse-transcription polymerase chain reaction (qRT-PCR) and microarray data from the GSE8361 dataset as potential markers for the early diagnosis of JIA, revealing new pathways and molecular targets for sJIA. Further studies with larger sample sizes and in-depth analysis are needed to confirm these results [28].

Ren et al. analyzed 70 active sJIA patients compared with 55 healthy controls to highlight differentially expressed genes (DEGs), hub gene sets, and patterns of immune system cell organization. The results showed 118 DEGs, of which 94 were upregulated (most: *CD177*, *OLFM4*, *ARHGEF12*, *MMP8*, *PLOD2*, *CEACAM6*, and *CEACAM8*) and 24 were downregulated (most: *TCL1A*, *ALOX15* and *HLA-DQB*). A hub gene set of eight upregulated genes (*ARG1*, *DEFA4*, *HP*, *MMP8*, *MMP9*, *MPO*, *OLFM4*, and *PGLYRP1*) was found, and none were downregulated. Functional enrichment analysis clearly showed that these eight hub genes were mainly connected with the complex tasks of neutrophils, playing a decisive role in the pathogenesis of sJIA, while macrophages, CD8+ T cells, and naïve B cells were relevant drivers of disease progression. *TPM2* and *GZMB* were identified as possible markers of positive short-term response to canakinumab treatment [29].

A summary of the abovementioned published studies on genetic biomarkers is shown in Table 1.

### 2.2. Specific Cellular Biomarkers in sJIA and MAS

#### 2.2.1. NK Cell Dysfunction in sJIA and MAS

Natural killer cells are large granular lymphocytes with cytotoxic activity that are crucial to the innate immune system, being a member of the rapidly growing innate lymphoid cell (ILC) family, which constitutes 5–20% of all circulating lymphocytes in humans and mediates antiviral and antitumor responses [30]. 

Innate lymphoid cells (ILCs) are the latest family of innate immune cells to be discovered from common lymphoid progenitors (CLPs). ILCs secrete signaling molecules and regulate both innate and adaptive immune cells. ILCs are mainly tissue-resident cells found in both lymphoid and non-lymphoid tissues, and not often in the blood [31].

ILCs are deeply embedded lymphocytes in tissues that do not express specialized antigen receptors for T and B cells. The study of ILCs has opened new perspectives for understanding immune regulation and maintenance of tissue homeostasis by the immune system. ILCs produce cytokines for various immune pathways expressed in relation to commensal agents and pathogens in mucosal barriers, while also stimulating adaptive immunity and adjusting inflammation in tissues, contributing to metabolic homeostasis, morphogenesis, remodeling, and tissue regeneration in direct association with the nervous system [32].

NK cells originate from hematopoietic stem cells that have recently been classified as ILCs in group 1 (ILC1s), comprising conventional NK (cNK) cells and subsets of “unconventional” NK cells. ILC1s are found in tissues and are also known as tissue-resident NK (trNK) cells, which are found in abundance in organs such as the liver, lungs, and uterus, whereas cNK cells circulate through the blood vessels. Although cNKs and ILC1s share interferon-gamma (IFN-γ) release when activated, these two categories of cells developed from different progenitors and have diverse tissue distributions and specific functions [32,33].

cNK cells have a much higher cytotoxic capacity and higher perforin expression compared to ILC1s, which have a low cytotoxic capacity but can release large amounts of IFN-γ, tumor necrosis factor alpha (TNF-α), and granulocyte–macrophage colony-stimulating factor (GM-CSF) [34,35].

NK cells’ numbers, growth, multiplication, and function are modulated and monitored by two pro-inflammatory cytokines: IL-6 and IL-18 [36,37].

Experimental studies showed that in mice with MAS, IL-6 levels were elevated in plasma and led to decreased perforin levels and granzyme B expression in NK cells, but without degranulation—phenomena that improved after targeted treatment with anti-IL-6. Similar attributes were found in patients with sJIA. Thus, IL-6 increases the inflammatory response and disrupts the function of NK cells, favoring the occurrence of MAS [38,39].

Among the first studies performed to inspect the functions of NK cells and their cytotoxicity in patients with sJIA complicated by MAS, Grom et al. used flow cytometry to investigate the expression of perforin in NK cells (CD56+/TCRαβ−), NK T cells (CD56+/TCRαβ+), and CD8+ cells. NK cells’ cytotoxic activity was quantified as measured after co-incubation with an NK-sensitive K562 cell line. The authors found two important categories of immunological dysfunctions: Some patients had reduced NK cell counts and activity, but slightly increased perforin expression in CD8+ and CD56+ cytotoxic cells. Other patients with sJIA and MAS had reduced NK activities associated with decreased perforin expression in all cytotoxic cells—a pattern similar to the perforin deficiency found in patients with familial hemophagocytic lymphohistiocytosis (fHLH). The authors’ conclusion was that in sJIA-MAS there is an NK cell dysfunction analogous to that in fHLH, which requires further investigation [40].

MAS has been described in association with many rheumatic disorders, but most often with sJIA. Clinical manifestations in MAS are similar to those of hemophagocytic lymphohistiocytosis (HLH)—a genetic disease accompanied by NK cell dysfunction. Villanueva et al. explored global NK cell dysfunction in JIA by studying peripheral blood mononuclear cells (PBMCs) from 40 patients with pauciarticular (*n* = 4), polyarticular (*n* = 16), and systemic (*n* = 20) subtypes of JIA. NK cells’ cytolytic function was determined after co-incubation of PBMCs with the NK-sensitive K562 cell line. NK cells (CD56+/T cell receptor [TCR]-αβ-), NK T cells (CD56+/TCR-αβ+), and CD8+ T cells were studied for perforin and granzyme B expression by flow cytometry. The results showed that NK cells’ cytolytic activity was much lower in sJIA patients than in other JIA groups or controls. The authors concluded that the subgroup of JIA patients who had not yet experienced any MAS event had low NK function and a lack of circulating CD56^bright^ cells, similar to defects found in patients with MAS and HLH. These aspects were particularly prevalent in the sJIA subtype, which is known to be strongly associated with MAS [41].

Due to the connection between IL-18 and the activity of NK cells triggered by this cytokine, de Jager et al. analyzed the relationship between high IL-18 plasma concentrations and the phenotype and function of NK cells in 15 patients with sJIA, the same number of patients with polyarticular JIA, and 10 healthy controls, via in vitro staining and functional assays. Using fluorescence microscopy, they investigated the binding of the IL-18 ligand, and the phosphorylation reactions of several MAP kinases and the IL-18β receptor (IL-18Rβ) were highlighted by the Western blot technique. The results indicated that IL-18 from the plasma of patients diagnosed with sJIA stimulated the activation of NK cells from healthy controls and bound to its cognate receptor. However, after IL-18 excitation, NK cells from sJIA patients failed to upregulate killer molecules such as perforin and IFN-γ, nor did they trigger the phosphorylation of receptor-activated MAP kinases. As a result of an alternative stimulation of NK cells with IL-12, induction of cytotoxic activity was found. However, the authors did not find any adjunctive results of the combination of IL-18 and IL-12 in sJIA. Immunoprecipitation of IL-18Rβ revealed that NK cells from sJIA patients failed to phosphorylate this receptor following IL-18 stimulation, indicating that NK cell dysfunction in sJIA is directly correlated with a defect in IL-18Rβ phosphorylation, which has important implications for the understanding of the pathophysiological mechanisms and future therapy of sJIA [42].

Zhou et al. used flow cytometry to investigate the NK cell count, cytotoxicity, and expression of perforin, granzyme B, interferon (IFN)-γ, and tumor necrosis factor (TNF)-α in peripheral blood samples from 72 children with active JIA (systemic, 25; polyarticular, 24; pauciarticular, 23) and 25 controls. The authors used specimens from 220 children with JIA (systemic, 84; polyarticular, 72; pauciarticular, 64) and 150 controls for KIR2DS2, KIR2DS4, KIR3DS1, KIR2DL1, KIR2DL2, KIR2DL3, and KIR3DL1 typing by polymerase chain reaction with sequence-specific oligonucleotide probes. The results of the research proved that sJIA patients had lower NK cell counts, cytotoxicity, and expression of perforin and granzyme B compared to controls, whereas those with pauci- and polyarticular JIA subtypes expressed higher levels of perforin and granzyme B. In patients with sJIA, NK cells generated lower amounts of TNF-α and higher amounts of IFN-γ than in the pauci- and polyarticular JIA groups. No significant disparities were found in KIR gene frequencies between JIA subgroups and healthy controls, except for KIR2DS4, which was lower in the sJIA group. Finally, the authors concluded that in sJIA there was a decline in the activity of NK cells, which secreted more IFN-γ and less TNF-α, and the incidence of KIR2DS4 was reduced [43].

Put et al. investigated NK-cell-specific transcriptional changes via RNA sequencing of highly purified NK cells from six patients with active sJIA and six healthy controls. The stimulatory cytokines—mainly for NK cells—were analyzed from plasma (*n* = 18), evaluating the phenotype and cytotoxic activity of NK cells on tumor cells (*n* = 10), as well as the production of IFN-γ (*n* = 8). For patients with sJIA, an altered gene expression profile of NK cells was found compared to healthy controls, with increased immunoinflammatory pathways, innate gene expression (TLR4 and S100A9), and decreased expression of immunoregulatory genes (i.e., interleukin 10 receptor, alpha subunit (IL10RA), and granzyme K (GZMK)). In patients with sJIA, increased IL-18 levels and a decreased IFN-γ/IL-18 ratio were found. NK cells had an imbalance between inhibitory and activating receptors, with reduced G1 receptor expression and increased expression of natural cytotoxicity triggering receptor 2 (NKp44). A reduction in granzyme K expression was observed in CD56^bright^ NK cells, along with defective IL-18-induced IFN-γ secretion and signaling. Hard-to-detect defects in the immune pathways governed by NK—such as the expression of granzyme K and the production of IFN-γ stimulated by IL-18—induce the immunoinflammatory dysfunctions that characterize sJIA [44].

The pathophysiology of sJIA and MAS still has many unclear elements, although it is known that there are high levels of IL-18 and reduced activity of NK cells in patients with active disease. Ohya et al. explored the phosphorylation of mitogen-activated protein kinase (MAPK) p38 and the enhancer of nuclear factor κ light chain of activated B cells (NF-κB) p65 in NK cells after being boosted by in vitro recombinant IL-18 (rIL-18) in 31 patients with sJIA and 6 healthy controls. The authors investigated the connections between clinical symptoms, serum IL-18 levels, and the strength of phosphorylation in NK cells. Patients were distributed in conformity with their disease activity into four groups: systemic characteristics (*n* = 8), chronic arthritis (*n* = 7), remission on medication (*n* = 10), and remission off medication (*n* = 6). The results showed that the phosphorylation of MAPK p38 and NF-κB p65 was more intense in the group of healthy patients than in those in remission without therapy, in remission on drugs, or with chronic arthritis, while there was a complete defect in phosphorylation in those with systemic manifestations. The serum concentration of IL-18 was highest in the group with systemic manifestations, followed by those with chronic arthritis, those in remission on medication, those in remission without medication and, finally, the healthy group. The authors concluded that the increased levels of IL-18 induce the phosphorylation defects of MAPK and NF-κB in NK cells, and that the inappropriate signaling of IL-18 in NK cells is directly related to the activity of sJIA [45].

#### 2.2.2. Macrophages in sJIA and MAS

Macrophages are known as cells equipped for the detection, phagocytosis, and destruction of bacterial agents, as well as other microorganisms that are harmful to the body. They are antigen-presenting cells to T lymphocytes and produce molecules (cytokines) that develop the inflammatory process by activating other cells. Two types of activated macrophages are known: classically activated macrophages, i.e., macrophages that encourage inflammation (M1); and macrophages alternately activated by phages, i.e., macrophages that decrease inflammation and encourage tissue repair (M2). The activated or polarized type (M1) secrete interleukins with pro-inflammatory action (e.g., TNF-α, IL-1 beta, IL-6, IL-12), chemokine (C-X-C motif) ligands 9 and 10 (CXCL9, CXCL10, etc.), and low amounts of IL-10, which promotes and modulates the immune response of Th1 helper cells (CD4+ cells or CD4-positive cells), i.e., Th1 lymphocytes [46].

Feng et al. investigated the roles played by different macrophage subtypes in the evolutionary stages of sJIA. The study included 22 children diagnosed with sJIA, divided into two groups: 12 patients with active disease, recently diagnosed and still without treatment, as well as 10 with inactive disease, who were monitored for two years. The control was a group of 10 children with orthostatic proteinuria. For all subjects, peripheral blood was analyzed by flow cytometry to highlight the following macrophage subtypes: M1 (CD14+CD86^+^CD80^+^), M2a (CD14^+^CD206^+^CD301^+^), M2b (CD14^+^CD206^+^CD86^+^), and M2c (CD14^+^CD206^+^CD163^+^), as well as IL-1β, IL-2, IL-4, IL-5, IL-6, IL-8, IL-10, IL-17, INF-α, INF-γ, and TNF-α. The authors found the M1 phenotype marker CD80, M2 phenotype marker CD163, and CD301 to be strongly expressed in children with active sJIA—a group in which most macrophages were M1 and M2a. In contrast, in the group with inactive disease, M2 was polarized predominantly into M2b and M2c. IL-6 had high levels in the group with active disease, while in the other group IL-4, IL-10, and IL-17 had high values. The authors concluded that M1 induces inflammation in active sJIA, while M2a almost simultaneously triggers inflammation inhibition, whereas M2b and M2c play major roles in inhibiting inflammation in inactive disease [47].

Macrophage polarization is a biological phenomenon through which these cells are able to follow certain functional programs as they are guided by signals from the microenvironment. Polarization can be caused by chemical stimulation or by stiffness substrates on which the macrophage is grown [48].

M2 macrophages can be polarized by the action of the cytokines IL-10, IL-13, and IL-4 which, thus activated, can inhibit inflammation and promote tissue repair, angiogenesis, and fibrosis. Recent studies have shown that M2 macrophages can be divided into different subtypes, such as M2a, M2b, M2c, and M2d, which have activities specific to the stage of the disease. M2a macrophages are polarized by IL-4 and IL-13, lowering the levels of IL-10 and transforming growth factor beta (TGF-β), participating in tissue regeneration and incorporation of pro-inflammatory molecules, thereby preventing the inflammatory response. M2b macrophages secrete IL-1, IL-6, IL-10, and TNF-α under the influence of immune complexes or lipopolysaccharides (LPSs), resulting in the activation of Th2 cells and the reducing inflammation. M2c macrophages are activated by IL-10, TGF-β, and glucocorticoids; they secrete IL-10 and TGF-β, which suppress the inflammatory response. The M2a subtype, in tandem with Th2 cells, participates in allergic reactions, while M2b is involved in immune regulation and M2c can secrete anti-inflammatory cytokines with a role in reducing inflammation, reshaping the matrix and tissues. M2d macrophages are polarized by IL-6 and adenosine, and are known as tumor-associated macrophages (TAMs). In sJIA, the cluster of differentiation 163 (CD163), arginase-1 (Arg-1), and chemokine (C-C motif) ligand 2 (CCL2) or monocyte chemoattractant protein 1 (MCP1) genes are closely related to M2 macrophages and are expressed on the surface of monocytes [49,50,51].

CD163 is a potent receptor specific to monocytes and macrophages for haptoglobin–hemoglobin complexes. CD163 is strongly induced by the anti-inflammatory cytokine IL-10, as well as by glucocorticoids and IL-6. CD163 easily induces TNF-like weak inducer of apoptosis (TWEAK) and is a receptor for a variety of bacteria and viruses. CD163 is widely used as a marker for alternately activated macrophages. CD163 +-activated macrophages have an immunomodulatory profile and are valued as anti-inflammatory markers [52].

CD163 helps regulate and extinguish inflammation, eliminates free hemoglobin, and is strongly expressed in myeloid cells in patients with sJIA and MAS [53].

#### 2.2.3. Neutrophils and Neutrophil Extracellular Traps

Neutrophils are cells that are part of the innate immune system, with an important role in the phagocytosis of pathogens; they originate from hematopoietic stem cells, have a short lifespan, and represent more than 50% of circulating white blood cells. The nuclear morphology of these cells gives them a phenotypic and functional heterogeneity. In addition to their ability to migrate, as well as their phagocytic and immunomodulatory functions, they can form neutrophil extracellular traps (NETs) in the form of a network of fibers consisting of cytoskeletal proteins, proteases, antimicrobial peptides, histones, and chromatin deoxyribonucleic acid [54,55,56].

It has been observed that in the early stages of sJIA’s onset, the innate immune cells with a defensive role—such as neutrophils and macrophages—are much more abundant in peripheral blood and play an essential role in the development of systemic inflammation. These cells produce several mediators involved in the pathogenesis of sJIA, of which IL-1 appears to play a pivotal role [57,58].

Ter Haar et al. investigated the role of neutrophils in 50 patients with sJIA receiving therapy with a recombinant IL-1 receptor antagonist (rIL-1Ra; anakinra). At the onset of the disease, the number of neutrophils increased significantly and was correlated directly with the pro-inflammatory factors, and after a few days of treatment with anakinra the neutrophil count returned to normal. RNA sequencing research has revealed an important adjustment of the inflammatory processes triggered by neutrophils in active disease patients, identical to the blood transcriptome analysis of patients with sepsis. Neutrophils from patients with active sJIA had a charged phenotype defined by increased reactive oxygen species (ROS) production, decreased L-selectin (CD62L) levels, and degranulation of secretory vesicles, which was completely changed after anakinra therapy in clinical remission patients. Those with a short period from the onset of sJIA had many neutrophils—especially immature ones—and a complete resolution of the disease under anti-rIL-1Ra, in contrast to those with a disease duration of more than one month, who had a normal number of neutrophils, but with an unsatisfactory response to anakinra treatment. These findings firmly suggest that neutrophils play an important role in sJIA’s onset and in the early inflammation stage, and that their counts and pro-inflammatory activity are influenced by IL-1 blockade [59].

To examine activated neutrophil subsets, the release of S100 alarmin, and gene expression signatures in patients with different spectra of sJIA activity, Brown et al. analyzed samples from 23 active sJIA patients and 22 children with clinically inactive disease (CID). Control samples were obtained from children undergoing evaluation for joint pain but found to have non-inflammatory disorders. The results indicated a higher percentage of CD16+CD62Llo neutrophils compared to controls. This subset of neutrophils was not observed in patients with CID or in those with active arthritis but without systemic features. CD16^+^CD62Llo neutrophils from sJIA-MAS patients showed increased nuclear hypersegmentation compared with CD16^+^CD62L^+^ neutrophils. Serum concentrations of S100A8/A9 and S100A12 were correlated very strongly with peripheral blood neutrophil counts. Whole-transcriptome analysis of highly purified neutrophils from children with active sJIA identified 214 differentially expressed genes (DEGs) compared with controls. In all samples, regardless of disease activity, increased inflammatory gene expression was found, including inflammasome components and S100A8. The authors’ conclusion was that in the case of activation of neutrophils in both active and inactive sJIA, a pro-inflammatory gene expression signature can be identified, indicating prolonged innate immune activation [60].

Known to be key phagocytic cells belonging to the innate immune system, neutrophils are endowed with the potential for oxidative and non-oxidative defense against pathogens. Their complex decision-shaping function, by which they instruct other leukocytes to adjust innate and adaptive immune responses, has recently been discovered. Under physiological conditions, they have a short life and are permanently set free by the bone marrow, starting from undifferentiated stem cells and going through multiple stages until maturation, in order to finally develop accelerated and intense immune responses. The human body needs many such cells during systemic inflammation in order to generate emergency granulopoiesis, but in excess they can cause tissue damage, as seen in sJIA. It is fundamental to better understand the activity and the types of neutrophils in the joints and blood of patients with sJIA. NET activation and release (NETosis) is a unique form of cell death in which neutrophils remove decondensed deoxyribonucleic acid (DNA), histones, cytokines, and granular proteins, and has never been studied directly in patients with sJIA. However, high serum histone levels in active sJIA, compared to inactive or healthy control patients, suggest increased NETosis, being well-correlated with sJIA disease activity. Increased high-mobility group box-1 (HMGB1) levels in patients with sJIA are associated with intensified NETosis, forming a positive feedback loop. Serum HMGB1 concentrations of NET molecules (including DNA without cells, myeloperoxidase (MPO)–DNA complexes, and α-defensin) were found to be increased in patients with adult-onset Still’s disease (AOSD) and sJIA, compared to healthy controls. NET molecules induce the release of HMGB1 and the production of IL-1β by monocytes, and as ligands of TLR4 they could be involved in activating the TLR signaling pathway in systemic arthritis [61,62,63]. 

In a very recent review, Kim et al. described the roles of neutrophils and the formation of NETs, which participate in the aggravation of inflammation in the pathogenesis of sJIA and AOSD. Activation of neutrophils and their surface receptors (including Fc receptors) by pathogen-associated molecular pattern (PAMP) or damage-associated molecular pattern (DAMP) molecules leads to their displacement in inflamed tissues and, subsequently, to the release of cytokines and chemokines with pro-inflammatory potential. Activated neutrophils and expelled mediators form a positive feedback loop that increases the recruitment of new neutrophils and exacerbates the inflammatory phenomena, on which the pathogenic mechanisms of AOSD and sJIA can be focused. Simultaneously, the activated neutrophils produce and release NETs and participate in the activation of macrophages in the inflamed area. At the same time, the levels of other DAMPs (e.g., HMGB-1, S100 proteins, LL-37, a human antimicrobial peptide from the cathelicidin group, and myeloperoxidase–DNA (MPO–DNA) complexes) increase in patients’ blood, accelerating systemic inflammation even more intensively. These novel results deepen the comprehension of the pathophysiological mechanisms involved in sJIA and AOSD, and can accelerate the patenting of neutrophil-associated biomarkers to confirm the diagnosis and likely course, as well as the development of original drugs to address the neutrophils and NETs [64].

#### 2.2.4. Platelets

In 2016, a group of experts analyzed 115 sJIA-MAS patient profiles, starting from laboratory data before the onset of MAS, followed by those corresponding to the onset of MAS, with a particular focus on the changes between the two key moments. They selected and ordered the most important laboratory tests with the most dramatic changes for the timely diagnosis of sJIA-MAS, the relevance of which was debated and cataloged at an expert consensus conference. At the end of the analysis, platelet count, ferritin level, and aspartate aminotransferase (AST), followed by white blood cell count, neutrophil count, fibrinogen (Fg), and erythrocyte sedimentation rate (ESR), were declared to be the most valuable laboratory parameters with the most important changes at the onset of sJIA-MAS [65].

In a recent review, Crayne et al. noted that laboratory features of MAS include pancytopenia, hyperferritinemia, fibrinolytic coagulopathy, and liver dysfunction [66].

At the 2020 American College of Rheumatology Congress, De Matteis et al. highlighted platelet count and ferritin as two significant parameters with high specificity and sensibility, respectively, to diagnose MAS-sJIA. The authors stipulated that these biomarkers could be practical prognosticators of the clinical outcomes and the effectiveness of the administered therapy [67].

The important roles of platelets and the MAPK pathway in the pathogenesis of sJIA provide a new perspective for exploring potential molecular targets for the treatment of sJIA, as Zhang et al. revealed by integrated bioinformatics analysis [28].

#### 2.2.5. Complete Blood Cell Count 

Currently, there is a consensus that sJIA and AOSD are similar diseases but emerge at different ages [68]. 

Children with sJIA are particularly susceptible to developing MAS. In a multinational, multicenter study that included 95 pediatric rheumatologists and hemato-oncologists from 33 countries, data collected from 362 patients were retrospectively investigated and included 22% with MAS at the onset of sJIA. Clinical manifestations in order of frequency were as follows: fever (96%), hepatomegaly (70%), splenomegaly (58%), central nervous system dysfunction (35%), and hemorrhages (20%). In terms of laboratory data, platelet counts and levels of liver transaminases, ferritin, LDH, triglycerides, and D-dimers were the only laboratory biomarkers showing a change of more than 50% between the pre-MAS consultation and the onset of MAS. Macrophagic hemophagocytosis was detected by bone marrow aspiration in 60% of patients. Approximately one-third of patients required admission to the intensive care unit (ICU), and the mortality rate was 8%. MAS remains a grave complication, as an important percentage of children require mandatory admission to an ICU or will die [69].

A group of experts statistically analyzed 982 potential criteria for 428 profiles of patients with MAS-sJIA and obtained the best results for 37 of them, analyzed in comparison with eight criteria from the literature. With a consensus of 82%, they obtained the final MAS classification criteria, validated with a sensitivity of 0.73 and a specificity of 0.99. These points of reference could contribute to a better insight into MAS in sJIA, to the discovery of new active and safe therapies, and to a better selection of patients for future clinical studies [15].

The fact that systemic inflammation in sJIA includes elevated ESR, C-reactive protein (CRP), white blood cell count, platelet count, ferritin, transaminases, aldolases, and D-dimers may help define disease activity; according to Lee et al., primary care must closely monitor patients with sJIA to detect early complications and unexpected drug reactions [7].

Some of the studies on cellular biomarkers discussed above are presented in Table 2.

### 2.3. Cytokines, Chemokines, and Other Biomarkers in sJIA and MAS 

#### 2.3.1. IL-1

The role of innate immune system involvement and the aberrant responses in the pathophysiology of sJIA have been documented in numerous clinical studies and publications [70].

The role of IL-1 in the pathogenesis of sJIA was first discovered by Virginia Pascual in 2005 [71]; since then, evidence has shown many other aspects of the involvement of this cytokine. Additional evidence includes clinical observations after the administration of biological agents blocking the IL-1 pathway via rIL-1Ra (anakinra) or prolonged blocking of IL-1 by the biological agent canakinumab [71,72,73,74,75].

Ling et al. highlighted that the flare versus quiescent signature in sJIA attests to the key role of IL-1 in the onset of the disease [76].

Several genetic alterations or mutations associated with dysregulated IL-1 activity and autoinflammatory disorders have been identified, and these findings have led to the successful use of IL-1 inhibitors in rheumatic diseases that were previously considered to be untreatable [77].

In a recent review on the role of IL-1 in general pathology, Kaneko et al. identified IL-1 as a key cytokine not only for cell-damage-related inflammation, but also for cell, tissue, and organ homeostasis in terms of overall pathology; thus, IL-1 has become an important molecular target for treating a wide range of diseases, such as autoinflammatory, autoimmune, and infectious diseases, malignant tumors, etc. [78].

Particular attention should be devoted to patients with recent-onset sJIA or pediatric Still’s disease—a syndrome with several clinical forms that may benefit from a treat-to-target strategy with a key role of IL-1 inhibition. Due to the heterogeneous and often difficult-to-treat nature of sJIA, the experience of expert teams is recommended [79].

At the onset of sJIA, it has been demonstrated that an early and correct treatment in the so-called “window of opportunity” by administering the use of IL-1 inhibitors—especially in steroid-naïve patients—can enable the interruption of the pathogenic cycle of the disease and induce a prompt and full remission. Ter Haar et al. conducted a prospective study of 42 patients diagnosed with sJIA at onset, but without an adequate response to non-steroidal anti-inflammatory drugs (NSAIDs), compared with 12 patients who did not have arthritis at onset, and both groups were followed for a mean period of 5.8 years. The first group received rIL-1Ra as monotherapy, as a targeted treatment. The results showed that the disease generally became inactive after 33 days, and after one year 76% of the patients had inactive disease, 52% of whom had not received any drug(s). It was observed that there was a correlation between the increased number of neutrophils at the beginning, the positive response after one month of treatment with rIL-1Ra, and the disappearance of symptoms one year after the initiation of the study. The monitoring of patients over a period of 5 years showed that 96% no longer had active disease, and 75% achieved inactive disease status without any medication. Articular and extra-articular damages were found in a small percentage (less than 5%) of cases, and only one-third of the patients required the administration of glucocorticoids. The authors also noted the effectiveness of therapy with rIL-1Ra in children with sJIA without arthritis at the onset. The use of rIL-1Ra as a first-line, short-term monotherapy may be a valuable option to interrupt the pathogenic cycle of sJIA at onset, in which IL-1 is the central component to be targeted [9].

Saccomanno et al. retrospectively analyzed the response to treatment with an IL-1 inhibitor (anakinra) for 62 patients diagnosed with sJIA over a 14-year period, including demographic, clinical, and laboratory data, as well as previous or concomitant therapies administered. The results of anakinra management were estimated by univariate and multivariable statistical analyses after one year of treatment, outlining the clinical patterns of patients with sJIA for whom IL-1 blockade may be a successful treatment. The authors recommend future in-depth studies on the efficacy of early therapy with IL-1 inhibitors, but also the detection of biomarkers that accurately predict the response to IL-1 or IL-6 antagonists [80].

Lainka et al. undertook a retrospective study on 202 patients diagnosed with sJIA and included in the German AID registry from 17 centers, of which 111 were children treated with IL-1 inhibitors, i.e., anakinra (ANA) (*n* = 84, 39 f) and/or canakinumab (CANA) (*n* = 27, 15 f). In the first year of therapy, 75/111 (ANA 55, CANA 20) patients were evaluated according to the Wallace criteria, and disease inactivation was achieved in 28/55 and 17/20, respectively, while remission over 6 months under medication was achieved in 13/55 and 7/20 patients, respectively. The clinical response to biological medication was maintained in most patients (ANA 54/80, CANA 20/27). The study acknowledged positive clinical results with both IL-1 inhibitors (anakinra and canakinumab), which were well-tolerated by patients and showed reasonable safety and efficiency when tested in a true clinical framework [81].

#### 2.3.2. IL-1β

Pro-inflammatory cytokines in the innate immune system—such as IL-1, IL-6, IL-18, and TNF-α—are directly involved in the inflammatory process of sJIA. For example, IL-1β—a pleiotropic pro-inflammatory cytokine—is excessively released by mononuclear cells in the blood and adjusts not only its own transcription, but also that of IL-6. The fundamental role of IL-1β in the pathogenesis of sJIA and the perpetuation of chronic inflammation has been demonstrated by stopping its activity after the administration of biological agents [82,83,84,85,86].

In recent decades, special efforts have been made to establish complex consensus-based plans for the early diagnosis and management of sJIA as an autoinflammatory disease. These strategies also involve the implementation of expensive treatment methods that directly target the main initiating and supporting cytokines (i.e., IL-1 and IL-6) of the chronic inflammatory process [81,87].

It has already been shown that if the initial autoinflammation with the disturbance of innate immunity is not stopped, the phenotype changes with the disruption of the elements of adaptive immunity will have evolutionary consequences in the form of destructive arthritis. If biological treatment is applied early—that is, when the “window of opportunity” is open—and there is financial possibility of long-term continuation, patients with sJIA will be more likely to be diagnosed with inactive disease and even go into remission.

It is believed that the mutation in the nucleotide-binding oligomerization domain-like receptor pyrin domain-containing protein 3 (NLRP3) inflammasome leads to overexpression of the cytokine IL-1β. The NLRP3 gene codifies the multimeric protein complex cryopyrin, which monitors the activation of caspase-1 which, in turn, catalyzes the cleavage of pro-IL-1β into IL-1β [88].

Inflammasomes are cytosolic multiprotein oligomers responsible for the activation of inflammatory responses as important parts of the innate immune system. NLRP3 inflammasome activation during infections requires two signals: one priming signal, and one activation signal. Once assembled, the NLRP3 inflammasome activation leads to the auto-cleavage of pro-caspase-1, which mediates the proteolytic process of pro-IL-1β, pro-IL-18, and the propyroptotic factor gasdermin D [89,90].

Studies on the pathogenesis of sJIA have shown the biphasic nature of events—at the beginning of systemic manifestations, the elements of innate immunity participate with the key role of the cytokine IL-1β; and in the second phase, chronic arthritis interacts with the adaptive immunity through the cytokine IL-17A. On this basis, IL-1 blocking treatment was initiated in the early stages of sJIA [91].

Brunner et al. investigated the long-term benefits and safety of canakinumab and the predictability of responses in 123 patients with sJIA with or without fever (70 with fever, and 52 without fever) at the start of therapy. Only 84 subjects (68.3%) remained in the study to completion—a mean period of 1.8 years. Clinical–biological sJIA-ACR 50 (American College of Rheumatology score = at least 50% improvement in disease activity) responses occurring by day 15 were the most representative predictor of clinical remission, according to the JADAS (Juvenile Arthritis Disease Activity Score), as well as of steroid discontinuation. The biological agent, as a monoclonal antibody that inhibits IL-1β and can decrease the levels of IL-6 and the hepatic synthesis of CRP and Fg, rapidly and persistently improved the clinical condition of patients with active sJIA, regardless of whether or not they had fever at the initiation of therapy [92].

MAS, as a potentially fatal complication of sJIA, may not respond to conventional doses of biological cytokine inhibitors requiring dose escalation. It has been shown that when MAS-sJIA is triggered, it can be treated with anakinra—i.e., with IL-1Ra—and increasing the dose can be beneficial for the patient. Another IL-1 inhibitor, canakinumab—which is a monoclonal antibody of IL-1β—has been reported to effectively treat MAS-sJIA that is refractory to conventional treatment. Kostik et al. retrospectively analyzed the data from the electronic medical records of an academic center with respect to the use of canakinumab in eight children (five girls) diagnosed with MAS-sJIA, with an average age of 8.5 years, during the period 2011–2020. Among these patients, five children developed MAS at the onset of sJIA, while three developed it during the treatment with canakinumab. MAS remitted in all children included in the research, but when the dose of canakinumab was not sufficient, or when MAS developed during the canakinumab therapy, the dose of the administered drug was increased in the short term (2–3 times the normal dose), without observed side effects, attesting to the efficacy and safety of increased doses of canakinumab in MAS-sJIA, but posing the need for additional studies [93].

#### 2.3.3. IL-6

IL-6 plays a special role in autoinflammatory diseases, and especially in sJIA, where it contributes to fever; increased acute-phase reactants (e.g., ESR, CRP, alpha-1 and alpha-2 globulins, Fg, haptoglobin, etc.), serum amyloid A (SAA), white blood cells, and platelets; and decreased red blood cells with chronic anemia. Elevated serum IL-6 concentrations have been found in several inflammatory diseases, such as AOSD and sJIA. IL-6 acts on B and T lymphocytes, hematopoietic progenitor cells, megakaryocytes, macrophages, hepatocytes, and neuronal cells [94].

In early research on the involvement of IL-6 in the pathophysiological mechanisms of sJIA, de Benedetti et al. evaluated serum IL-6 levels in 25 patients with systemic-onset juvenile arthritis using the B9 hybridoma cell line, finding significantly increased concentrations during active disease—correlated with the extent and severity of joint involvement and platelet counts—but not during remission. This study highlighted the important role of IL-6 in the pathogenesis of sJIA [95].

IL-6 levels are elevated in both serum and synovial fluid in patients with sJIA, directly related to fever, growth deficits, anemic syndrome, and thrombocytosis [96,97].

In a study on early changes in inflammatory gene and protein expression in sJIA managed with anti-IL-1β human monoclonal antibody therapy, Brachat et al. measured gene expression in febrile sJIA patients and matched healthy controls. Transcriptional response was assessed by changes in gene expression from baseline to day 3 using the adapted sJIA-ACR 50 response. The pro-inflammatory cytokines IL-6 and IL-18 were measured up to day 197. The strongest clinical response was seen in patients with higher baseline expression of dysregulated genes and a strong transcriptional response on day 3. IL-6 decreased on day 3 (≥8-fold decrease) and remained suppressed. IL-18 decreased at day 57 (≥1.5-fold decrease). The authors identified an important response signature to canakinumab for the majority of sJIA patients, with the transcript levels for sJIA overexpressed genes quickly returning to normal—including those associated with the IL-1 and IL-6 signaling pathways—as well as a marked decrease in transcription of the gene encoding the target, i.e., IL-1β, meaning that this treatment interrupts a positive feedback loop between IL-1β signaling and IL-1β production. Canakinumab treatment in sJIA led to downregulation of innate immune response genes and downregulation of IL-6, but also decreased the clinical symptoms, paving the way for further research [98].

IL-6 is a cytokine with a complex role in local and systemic inflammation in the pathogenesis of sJIA, as well as in complications of the disease. As it is not known exactly how it works, the choice of IL-6 inhibitors should be made in the context of genetic and biological factors, as well as the type and phase of the disease [99].

Tocilizumab (TCZ), a humanized monoclonal antibody against the human IL-6 receptor, has radically altered the course of patients with sJIA and MAS. However, MAS may occur in patients with sJIA under TCZ therapy. Thus, it could be concluded that blocking IL-6 with TCZ is not sufficient to inhibit the pathogenic mechanism of sJIA and MAS [100,101].

Bielak et al. reported the results of treatment with biological agents for 200 patients with sJIA from the German Registry of Autoinflammatory Diseases (AID), of whom 46 received TCZ therapy as an IL-6 blocker, causing the disease to become inactive and/or enter remission after one year from the initiation of the treatment [102].

In a study on the action of TCZ in sJIA-MAS, Shimizu et al. combined expert consensus with an analysis of clinical and laboratory data for 12 patients treated with TCZ, compared with 18 sJIA-MAS patients without treatment. Patients who received TCZ were less febrile and had significantly lower levels of ferritin, triglycerides, and CRP than the control group. TCZ-treated patients with MAS associated with sJIA were less likely to be classified as having MAS based on the MAS classification criteria, due to the absence of fever and lower ferritin levels compared to untreated patients, leading to the conclusion that TCZ could alter the clinical and laboratory features of MAS associated with sJIA. Thus, when patients with sJIA are evaluated during TCZ treatment, the criteria for MAS cannot be applied, and great care must be taken not to fall into the trap of underdiagnosing MAS [103].

Aiming to elucidate the immune profile of sJIA to identify clinical biomarkers correlated with new immune mechanisms, Qu et al. analyzed plasma samples from 21 patients diagnosed with sJIA, compared with 60 age- and sex-matched healthy controls, via a highly sensitive proteomic immunoassay and analysis of canonical pathways associated with cellular functions. The authors investigated well-known sJIA biomarkers such as IL-6, IL-18, and S100A12, which have been shown to be elevated during active sJIA compared to healthy controls. IL-18 was the only one of these biomarkers with elevated levels in inactive sJIA compared to healthy controls. Other novel factors—such as CASP8, CCL23, CD6, CXCL1, CXCL11, CXCL5, EIF4EBP1, KITLG, MMP1, OSM, SIRT2, SULT1A1, and TNFSF11—were found to be differentially expressed in active and/or inactive sJIA compared to the control group. HMGB1 levels were found to be higher in active than in inactive sJIA. The authors’ results contribute to a deeper understanding of the immune mechanisms in active sJIA, for new diagnostic and therapeutic plans of action [104].

#### 2.3.4. IL-10

Interleukin 10 is a pleiotropic cytokine with various effects in immunoregulation and inflammation, secreted by almost all cells participating in immune defense, such as macrophages, mast cells, neutrophils, basophils, eosinophils, dendritic cells, B cells, and the various subtypes of T cells [105].

The main functions of IL-10 are performed through autocrine and paracrine mechanisms, which primarily give it an anti-inflammatory, inhibitory, or autoregulatory role through a negative feedback on inflammatory processes. IL-10 suppresses harmful inflammatory responses by preventing antigen presentation by dendritic cells and inhibiting the stimulation and penetration of macrophages in the lesion area, with the subsidiary consequence of reducing the expression of pro-inflammatory cytokines. Expanding the research areas for new cell-based therapies that benefit from the knowledge of IL-10 signaling pathways could bring important benefits to patients with chronic inflammatory phenomena and fibrotic consequences [106].

In a translational study using a comparative murine model of sJIA and samples from patients diagnosed with sJIA, Imbrechts et al. investigated whether the pathophysiological mechanisms of sJIA could be caused by defects in IL-10—a cytokine known to be crucial in controlling inflammation. The authors used a translational approach with an sJIA-like murine model, compared with samples from patients with sJIA. Freund’s complete adjuvant (CFA) containing heat-killed mycobacteria (1.5 mg/mL) was injected into IFN-γ-deficient BALB/c mice, as the corresponding wild-type (WT) mice are known to develop only a mild and transient inflammatory reaction. Diseased IFN-γ-deficient mice showed defective IL-10 production in CD4^+^ regulatory T cells, CD19^+^ B cells, and CD3-CD122^+^CD49b^+^NK cells, with B cells as the main IL-10 generators. Neutralization of IL-10 in WT mice generated a chronic immune inflammatory condition with an identical clinical and hematological picture to sJIA. The authors found plasma levels of IL-10 to be remarkably low compared to pro-inflammatory mediators in patients with sJIA, and CD19^+^ B cells from these patients showed decreased production of IL-10 both ex vivo and after stimulation in vitro. The authors concluded that neutralization of IL-10 in WT mice by CFA transformed a transient inflammatory reaction into a chronic disease and found cell-specific IL-10 imperfections in both mice and patients with sJIA, along with insufficient production of IL-10 to counterbalance the pro-inflammatory cytokines, indicating that the faulty production of IL-10 could contribute to the pathogenesis of sJIA [107].

Peng et al. investigated IL-10 concentrations in 21 patients with sJIA, compared with 35 patients with febrile illnesses suspected to be sJIA. The results showed that subjects with confirmed sJIA had significantly higher levels of IL-10 compared to those with other febrile illnesses; at the same time, serum concentrations of IL-10 were much higher in patients with active sJIA compared to those with inactive sJIA, with a direct relationship with other activity markers such as ESR, CRP, serum ferritin, and IL-6. Concomitantly, serum IL-10 levels at the time of diagnosis were observed to be much higher in sJIA patients with a non-monocyclic course than in those with a monocyclic pattern. IL-10 concentrations were more valuable in distinguishing monocyclic from non-monocyclic patterns in sJIA outcomes, in comparison with CRP, ESR, serum ferritin, and IL-6. The study showed that IL-10 levels were much higher in sJIA, leading to differentiation from other febrile diseases and a correct diagnosis, and were associated with the type of activity and disease progression. The authors proposed that serum IL-10 should be considered a reliable clinical marker in the diagnosis and prognosis of sJIA [108].

#### 2.3.5. IL-18

IL-18, which belongs to the IL-1 cytokine family, is initially inactive and is presented as an integral membrane protein, and then under the action of caspase-1 it transforms into a form of active cytokine to induce IFN-γ. IL-18 can be found as a constitutive protein in almost all human cells and in healthy animals; it is buffered by its natural inhibitor—an IL-18-binding protein called IL-18BP, which has a high affinity and regulates IL-18 activity [109]. 

Lotito et al., in a group of 50 patients with JIA (13 systemic, 13 polyarticular, 24 oligoarticular) and 25 controls, were among the first to investigate the levels of IL-18 in synovial fluid (SF) and serum, in correlation with the activity and severity of the disease. The concentrations of the studied cytokines (i.e., IL-1, IL-1Ra, IL-6, and IL-18) were higher in the sera of patients with JIA than in controls. IL-18 levels were as high in serum as in SF and were positively correlated with IL-1, IL-1Ra, IL-6, and other disease activity parameters (i.e., CRP, number of active joints, and radiological score). However, the levels of IL-18 and IL-6 in SF and serum were much higher in patients with sJIA than in those with other types of the disease. The authors concluded that IL-18 is involved in the pathophysiology of JIA, reflects the severity of the disease, and could be a target for the treatment of arthritis [110]. 

Shimizu et al. studied serum levels of the cytokines IL-18, IL-6, neopterin, and TNF-α receptor types I and II, along with ferritin concentrations, in 5 subjects with sJIA complicated by MAS (sJIA-MAS), 10 patients with Epstein–Barr-virus-induced hemophagocytic lymphohistiocytosis (EBV-HLH), 22 patients with KD, and 28 healthy controls. Biological data were compared with clinical symptoms of sJIA-MAS. Serum samples revealed significantly higher levels of IL-18 in patients with sJIA-MAS compared to patients with EBV-HLH or KD. In contrast, IL-6 levels in KD subjects were significantly higher than those in EBV-HLH or sJIA-MAS subjects, but serum neopterin levels in EBV-HLH patients were higher than those in sJIA-MAS or KD patients. A positive correlation was observed between serum levels of IL-18 and parameters of disease activity such as CRP, ferritin, and LDH. Serum IL-18 also remained elevated in patients with sJIA in the inactive stage of the disease, while clinical parameters and other cytokines normalized. The serum level of IL-18 can be considered to be a biomarker of sJIA activity, and monitoring its profile could be beneficial for distinguishing MAS/HLH and estimating sJIA disease activity [111].

In another study, Shimizu et al. measured serum IL-6/IL-18 levels in 76 patients with sJIA, including 15 with MAS, and compared them with clinical symptoms. Depending on the IL-6/IL-18 serum concentrations, two distinct subsets were detected: The IL-6-dominant subset was more likely to develop much more severe joint disease. The IL-18-dominant subset included a larger number of patients who developed MAS and in whom IL-18 concentrations during the active phase were significantly higher, compared to those without MAS. The minimum serum level found for IL-18 was 47,750 pg/mL, which predicted the development of MAS. IL-18 could be involved in the pathophysiology of MAS, and serum concentrations > 47,750 pg/mL could be useful in predicting the initiation of MAS [112].

Xia et al. investigated whether IL-18 could be a valuable biomarker in the diagnosis of sJIA, comparatively investigating 23 patients with sJIA, 20 patients with acute lymphoblastic leukemia (ALL), 18 patients with severe infections (SIFs), 26 patients with Kawasaki disease (KD), 18 patients with JIA, and 25 healthy controls. Serum levels of IL-6, IL-18, S100A8, and S100A9 were measured. The results indicated significantly higher serum IL-6 concentrations in all patient groups, without significant differences between them, compared to healthy controls. S100A8 was significantly increased in sJIA vs. ALL, JIA, and healthy controls, but was not significantly different vs. the SIF and KD groups. S100A9 in sJIA was greatly increased compared to ALL and healthy controls, but not significantly different from SIF, KD, and JIA. The levels of IL-18 in sJIA were much higher than in groups with febrile diseases, allowing the authors to conclude that serum IL-18 could be a biomarker in the differential diagnosis of sJIA compared to other febrile diseases [113].

As an IFN-γ-stimulating production factor, IL-18 is a cytokine that plays an important role in Th1 and NK cell activation, but also in Th2, IL-17-producing γδ T cells, and macrophage activation in the processes of intracellular defense against bacteria and some viruses. IL-18 shares the same signaling pathway as IL-1 to initiate NF-κB and sets in motion some inflammatory mediators (e.g., adhesion molecules, chemokines, and Fas ligand (FasL)). IL-18, as a pleiotropic cytokine, plays an important role in various infectious, metabolic, and inflammatory diseases. IL-18 flows in the range of tens of nanograms/mL. Particularly high IL-18 concentrations in the sera of patients with active sJIA have been reported in several studies and correlated with elevated serum ferritin levels, predictive of MAS. sJIA and AOSD are characterized by high serum IL-18 concentrations and could be treated by IL-18BP [114].

Kudela et al. provided additional evidence in a study of 30 adult patients diagnosed with AOSD and 20 children diagnosed with sJIA with serum IL-18 levels up to 1000-fold higher during active disease compared to other rheumatic subtypes, proposing IL-18 as a biomarker for disease activity [115].

Yasin et al. investigated the total levels of IL-18, CXCL9, and S100 proteins in 40 patients with sJIA, comparing them between subjects to detect disease activity and history of MAS. Total IL-18 was greatly increased in patients with active sJIA and remained persistently elevated even in the vast majority of patients with inactive disease. Subjects with a history of MAS presented much higher concentrations of IL-18 compared to those who had never had MAS. Total IL-18 could predict disease activity and history of MAS. A moderate correlation was noted between IL-18 and CXCL9, as well as between S100A8/A9 and S100A12, being more pronounced for ferritin and, generally, for those with active disease. Elevated IL-18 may signal increased sJIA activity or the development of MAS [116].

Krei et al. conducted a systemic review of a total of 14 studies that included all HLH subtypes (and MAS subtypes) in both children and adults, investigating the role of IL-18 as a potential biomarker for the diagnosis and monitoring of HLH/MAS. Serum IL-18 was found to be elevated in both primary and secondary HLH (>1000 pg/mL), with a significantly higher level in MAS (IL-18 > 10 000 pg/mL) compared to other inflammatory conditions and healthy patients; therefore, serum IL-18 levels may differentiate between HLH subtypes and other inflammatory conditions. The authors concluded that IL-18 is a potential biomarker for HLH/MAS but is not currently part of the diagnostic criteria. IL-18 may help to differentiate between HLH subtypes and other inflammatory conditions. The potential of IL-18 to distinguish MAS from sJIA is more ambiguous, as IL-18 levels > 100,000 pg/mL were described in sJIA patients both with and without MAS [117].

Mizuta et al. investigated the significance of serum IL-18 concentrations for the diagnosis and prediction of sJIA’s progression; 116 patients with sJIA, 151 with other diseases, and 20 healthy controls were studied. IL-18 was measured in 41 patients with sJIA from the active period until remission. The minimum serum IL-18 concentration required for distinction from other diseases was 4800 pg/mL. IL-18 was steadily decreased during inactivity and remission in patients with monocyclic evolution. In contrast, in patients with polycyclic evolution, serum IL-18 concentrations were increased during the active period and normalized during the inactive phase. The cutoff value of serum IL-18 for remission in sJIA was 595 pg/mL. The authors noted that serum IL-18 levels > 4800 pg/mL may be useful in differentiating between sJIA and other autoimmune/autoinflammatory diseases; additionally, serum IL-18 levels may be useful in predicting progression and remission in sJIA [118].

Other recent studies have reported IL-18 as a predictive biomarker for the occurrence of MAS, along with the correlation of its high serum levels with the clinical parameters of MAS activity in patients treated with TCZ [103,119].

IL-18BP is abundant in the bloodstream under normal conditions and in many pathological disorders, stopping the negative systemic pro-inflammatory impact of IL-18. Severe clinical manifestations of sJIA are dependent on the levels of free IL-18 being elevated in the bloodstream, along with the imbalance between IL-18 and IL-18BP. The disequilibrium of IL-18/IL-18BP in favor of free-circulating IL-18 is known for its important role in the pathogenesis of sJIA, AOSD, and the severe complication of MAS. The use of recombinant IL-18BP in patients with AOSD or sJIA with MAS has shown promising results, making free IL-18 a biomarker and a therapeutic target in these diseases [120].

Recent findings support a pattern in which patients with high serum IL-18 levels (associated with pro-inflammatory activated neutrophils in the bloodstream) but low/normal CXCL9 levels are likely to respond well to IL-1 blockade. However, patients with sJIA who have low IL-18 (i.e., weak neutrophil marks) and low CXCL9 levels are unlikely to respond to IL-1 blockade, while patients with high IL-18 but also high CXCL9—reflecting the activation of IFN-γ (i.e., IL-18/CXCL9 ratio remains lower)—have a high risk of both MAS and failure of therapy [121].

Several studies have indicated that CXCL9, IL-18, neopterin, and the soluble TNF receptor (sTNFR) type II (sTNFR-II) could be useful in predicting MAS in patients with active sJIA. IFN-γ and the IFN-induced mediators are elevated during active MAS, and high blood levels of CXCL9 are predictive of future MAS. However, recent research has shown the opposite, where most patients with sJIA, despite significant increases in CXCL9, never developed MAS; this can be explained by the multiplex platform used in the laboratory determinations. Therefore, given these uncertainties, the use of CXCL9 and IL-18 to predict the outcome of IL-1 blocking treatment requires further study.

#### 2.3.6. IFN-γ

IFN-γ is mainly regulated by NK cells and natural killer T (NKT) cells as a reaction of the innate immune system, and by CD4^+^Th1 and CD8^+^ cytotoxic T lymphocyte (CTL) effector T cells after the antigen-specific (i.e., adaptive) immunity develops [122].

IFN-γ attaches to and stimulates the specific receptors of antigen-presenting histiocytes and dendritic cells in tissues, causing the synthesis and release of CXCL9 and CXCL10 chemokines, which bind CTLs in the peripheral blood; when activated, these CTLs move to the site of inflammation, where they release more CTL-derived IFN-γ which, in turn, increase the inflammatory response [123].

On the other hand, IFN-γ, as a pro-inflammatory cytokine, plays an important role in regulating hematopoietic stem cells (HSCs) in physiological conditions. IFN-γ actively plays a special pathogenic role in various diseases that harm hematopoiesis, including HLH, aplastic anemia, liver cirrhosis, etc. Increased serum IFN-γ levels have been shown to negatively influence HSC balance by overstimulating differentiation and self-renewal until exhaustion of HSCs [124].

Antigen-presenting cells activate T lymphocytes and NK cells, which secrete IFN-γ which, in turn, activates monocytes and macrophages. IFN-γ-activated M1-type macrophages secrete large amounts of pro-inflammatory cytokines, including IL-6, IL-12, and IL-23, as well as IP-10 chemokines and monokines that recruit polarized Th1 lymphocytes and NK cells. At the same time, the continuous stimulation produced by IFN-γ activates the macrophages that participate in the phenomenon of hemophagocytosis [14].

There are studies that show that IFN-γ plays a key role in triggering primary hemophagocytic lymphohistiocytosis (pHLH) and MAS, because serum levels of IFN-γ and of the IFN-γ-induced chemokines are significantly elevated. Thus, the clinical manifestations of pHLH and MAS can be stopped by therapy with monoclonal antibodies directed against IFN-γ. The use of emapalumab—an anti-IFN-γ monoclonal antibody—for the treatment of patients with pHLH symptoms and disease that is refractory, recurrent, progressive, or intolerant to conventional therapy has been approved by the Food and Drug Administration (FDA) [125,126,127].

Put et al. undertook research that aimed to determine the role of IFN-γ in the pathogenesis of sJIA and HLH by discovering a profile of IFN-γ and its connection with other cytokines; 10 patients with active sJIA, 10 patients with inactive sJIA, and 5 patients with HLH—of whom 3 had associated sJIA-MAS—were included in the study, along with 16 healthy controls. The authors investigated the cytokines, the IFN-γ-induced genes, and the proteins from patients’ plasma and peripheral blood mononuclear cells (PBMCs), as well as the lymph node biopsies taken from a patient during two sJIA and MAS episodes. IFN-γ responses were also investigated in healthy donors’ PBMCs, primary fibroblasts, and endothelial cells. The results showed that plasma IFN-γ, IL-6, and IL-18 levels were increased in active sJIA and HLH. The concentrations of IFN-γ and IFN-γ-induced proteins (i.e., IP-10/CXCL-10, IL-18BP, and indoleamine 2,3-dioxygenase) in patients with HLH were significantly higher than those in patients with active sJIA, but the free IL-18 and IL-18/IFN-γ ratios were higher in active sJIA than in HLH. HLH PBMCs demonstrated hyposensitivity to IFN-γ in vitro compared with PBMCs from control subjects and those with sJIA. Both fibroblasts and endothelial cells expressed proteins induced by IFN-γ in situ—as evidenced by staining samples from the lymph nodes of a patient with MAS—and in vitro upon stimulation with IFN-γ. The authors found recognizably different cytokine profiles, with very high plasma levels of IFN-γ and IFN-γ-induced proteins, in patients with active sJIA and HLH/MAS. Histiocytes, endothelial cells, and fibroblast cells, along with PBMCs, may facilitate a distinct plasma IFN-γ profile. The high concentrations of IFN-γ, compared to those of IL-18, may indicate a presumptive progression of MAS in sJIA [128].

Bracaglia et al. hypothesized that IFN-γ would be the pivotal mediator in murine pHLH models and that there would be a similarity between primary and secondary HLH—including MAS—and investigated the involvement of the IFN-γ pathway in MAS by measuring IFN-γ levels and induced chemokines, along with their correlation with laboratory data, in patients with sJIA-MAS and in a murine MAS model. Serum levels of IL-1β, IL-6, IFN-γ, and the IFN-γ-induced chemokines CXCL9, CXCL10, and CXCL11 were evaluated in patients with sec-HLH (*n* = 11) and in patients with sJIA (*n* = 54), of whom 20 had active MAS at sampling. The results showed that IFN-γ and the IFN-γ-induced chemokines were significantly increased in active MAS and sec-HLH compared to active sJIA without MAS. In patients with active MAS, serum ferritin, alanine transferase, neutrophils, and platelet counts were significantly correlated with serum IFN-γ and CXCL9 levels. In murine MAS, serum ferritin was significantly correlated with CXCL9 mRNA concentrations in the liver and spleen. The authors concluded that elevated levels of IFN-γ and IFN-γ-induced chemokines, in combination with other severely altered laboratory parameters in MAS, support the significant involvement of IFN-γ in MAS [129].

In another study, Bracaglia et al. analyzed the levels of IFN-γ, CXCL9, CXCL10, and IL-18 in 24 patients with active sJIA and in 37 samples from MAS patients with different degrees of disease severity and under different treatments at the time of the study. The data obtained showed that the levels of IFN-γ, CXCL9, CXCL10, IL-18, neopterin, and soluble interleukin-2 receptor alpha chain (sIL2RA or sCD25) were significantly increased in MAS compared to active sJIA without MAS and were correlated with laboratory parameters of disease severity, except for IL-18, whose levels were only available for some of the samples. In patients with active sJIA without MAS, levels of IFN-γ-induced chemokine (i.e., CXCL9 and CXCL10) were significantly higher in those with a positive history of MAS compared to patients without a history of MAS. In conclusion, the increase in neopterin and CXCL9—under the influence of IFN-γ, and in direct connection with laboratory parameters—was indicative of the role of IFN-γ in MAS. Higher increases in serum CXCL9 and CXCL10 levels in patients with a history of MAS compared to patients who had never had MAS emphasized the activation of this pathway even in the absence of obvious clinical MAS, suggesting that these two chemokines could predict the risk of MAS. Concentrations of neopterin, IFN-γ, CXCL9, CXCL10, sCD25, and IL-18 can be used as biomarkers to discriminate MAS from active sJIA [130].

MAS and sec-HLH are hyperinflammatory disorders that can be triggered by a CS. Prompt detection and early therapy are necessary to avoid the unfortunate consequences of these diseases’ high mortality. As IFN-γ is already known to play a major role in triggering sec-HLH and sJIA-MAS, De Matteis et al. performed a study on 35 patients with sec-HLH, 21 patients with sJIA-MAS, and 25 patients with sJIA at three different intervals: active disease (T0), 7–10 days after initiation of therapy (T1), and when the disease was no longer active (T2), i.e., 1–3 months after onset. Serum biomarkers related to IFN-γ (i.e., CXCL9, CXCL10, and neopterin) from 265 samples were investigated. The results of the study showed that the concentrations of CXCL9, CXCL10, and neopterin at T0 were much higher in patients with MAS and sec-HLH compared to those with sJIA, while they were progressively reduced at T1 and normalized at T2. A more rapid decrease was observed for CXCL9 than for neopterin, similar to the drop in routine laboratory data. The study confirmed that platelet count and ferritin were the most distinctive laboratory parameters with high specificity and sensitivity for sJIA-MAS. At the same time, elevated LDH levels, which are not included in the 2016 classification criteria for sJIA-MAS, may be useful in predicting the occurrence of MAS. The results revealed increased levels of IFN-γ-related biomarkers in patients with MAS and sec-HLH, which could be useful together with traditional laboratory parameters for diagnosis, clinical follow-up, and response to therapy [67].

Guo et al. performed a retrospective cohort study of 149 patients with sJIA, of whom 27 had 31 episodes of MAS. Clinical and laboratory parameters of patients with sJIA-MAS were investigated and compared with those without MAS. Clinically, a high percentage of patients with MAS at onset did not have fever, arthritis, or central nervous system dysfunction; 35% of MAS patients had hypotension without shock, and 22.6% of patients had gastrointestinal symptoms. Subjects with MAS and hypotension had a higher rate of ICU hospitalization; they presented more frequently with arthritis, serositis, pneumonia, and gastrointestinal symptoms, accompanied by higher white blood cell and neutrophil counts, superior serum bilirubin concentrations, and lower total serum protein levels. The platelet count, LDH, and aspartate aminotransferase (AST), together with the ferritin/ESR ratio of approximately 20.0, had high sensitivity and specificity in the early diagnosis of MAS. The association between IFN-γ > 17.1 pg/mL and IL-10 > 7.8 pg/mL appears to be a valuable cytokine prototype to confirm the onset of MAS.

The sudden onset of arterial hypotension, the ferritin/ESR ratio, and the pattern of elevated IFN-γ and IL-10 cytokines are important parameters in predicting the early onset of sJIA-MAS [131].

#### 2.3.7. TNF-α

TNF-α, recognized as TNF superfamily member 2 (TNFSF2), is a multifunctional cytokine with a well-determined role in innate and adaptive immunity, as well as in the normal physiology of the immune system. Abnormally high and sustained production of TNF-α is correlated with pathological manifestations of inflammatory diseases such as sepsis and chronic ADs. Physiologically, TNF-α is a crucial component for a normal immune response and can be considered a key factor in the development of infectious and autoimmune pathologies [132].

In the pathogenesis of MAS, it has been proposed that macrophages produce several cytokines, including TNF-α and pro-inflammatory interleukins (e.g., IL-6, IL-1, and IL-18), which unleash a cascade of inflammatory pathways and, ultimately, create a CS. TNF-α can be considered a key factor in the development of infectious and autoimmune pathologies. With advances in the development of many diagnostic tools to identify MAS in sJIA as well as other forms of MAS/sHLH, early management with cytokine-targeted therapies will likely save the lives of many patients. TNF-α, when produced in excess, participates in the spread of inflammatory phenomena by activating and concentrating fibroblasts, causing joint erosion, fibrosis, and joint deformity in rheumatic diseases. There are already five TNF-α receptor blockers (i.e., infliximab, adalimumab, etanercept, certolizumab pegol, and golimumab) currently used in the treatment of several inflammatory diseases, including in children [133,134].

Hügle et al. conducted a retrospective study of 32 patients for a period of 6 years, considering demographic and clinical data as well as the presence and titers of antinuclear antibodies (ANA) and rheumatoid factor (RF) at diagnosis, from the AID-Net database—a German registry and biobank that gathers information and biomaterials from patients with autoinflammatory syndromes, including periodic fever syndromes and sJIA; 8/32 patients had ANA titers ≥ 1:80 at diagnosis, 22/32 patients had rising ANA titers with titers ≥ 1:80 at their last visit, and 10/32 patients had a positive RF at least once during follow-up, compared to 0/32 at diagnosis. The authors concluded that a proportion of patients with sJIA developed increased ANA titers and positive RF over time, indicating the potential role of lymphocyte activation and autoimmune processes in this primarily autoinflammatory condition, independent of TNF antagonist treatment in sJIA [135].

Shimizu et al. measured the ratio between the levels of soluble TNF receptor (sTNFR) type II (sTNFR-II) and type I (sTNFR-I)—i.e., (sTNFR-II/I)—in 117 patients with sJIA, including 29 patients with MAS, 15 with EBV-HLH, 15 with KD, and 28 healthy controls, in correlation with the activity and severity of the disease. At the same time, they determined the serum levels of IL-18 in patients with EBV-HLH compared to MAS. The results showed that the ratio of sTNFR-II/I had a significantly higher level in patients with MAS and EBV-HLH compared to patients with active sJIA and KD. Serum IL-18 concentrations were also significantly increased in patients with MAS in comparison with EBV-HLH. Serum IL-18 and sTNFR-II/I could be used as biomarkers for the diagnosis of MAS and the differentiation between MAS and EBV-HLH [136].

Irabu et al. studied the serum concentrations of neopterin, IL-18, chemokine (C-X-C motif) ligand 9 (CXCL9), sTNFR-I, and sTNFR-II in a cohort of 36 patients with sJIA-MAS, including 12 patients under management with TCZ. The serum ratio of sTNFR-II/I was compared with the clinical characteristics of MAS. The results showed that the levels of all serum cytokines studied at the diagnosis of MAS were much lower in the TCZ group compared to those without this treatment, even if the serum sTNFR-II/I ratio at the diagnosis of MAS was similar between the compared groups. The serum ratio of sTNFR-II/I, which was elevated in sJIA-MAS patients, was positively correlated with disease activity, with cutoff values of 0.9722 and 4.71, respectively. The authors concluded that the serum sTNFR-II/I ratio could be a useful biomarker for assessing disease activity in sJIA-MAS, but also a predictive biomarker for the onset of MAS in the sJIA active phase—even in patients under TCZ [137].

Mizuta et al. comparatively studied the cytokines involved in the onset of MAS in order to detect the serum biomarkers for early prediction of MAS in different childhood rheumatic pathologies. Serum concentrations of neopterin, IL-6, IL-18, sTNFR-I, and sTNFR-II were investigated via the ELISA technique in 12 patients with SLE, including 5 with MAS; 12 patients with juvenile dermatomyositis (JDM), including 4 with MAS; 75 patients with KD, including 6 with MAS; and 179 patients with sJIA, including 43 with MAS. The results were analyzed in the context of the clinical symptoms of MAS. It was observed that the serum concentrations of neopterin, IL-18, and sTNFR-II were much higher during MAS compared to the active phase in all patients and all conditions studied. Serum concentrations of sTNFR-I were much higher during MAS compared to the active phase only in patients with SLE, KD, and sJIA. Depending on the disease, the curve analysis revealed the following cytokines at the highest levels: serum sTNFR-I for SLE, serum IL-18 for JDM, and serum sTNFR-II for KD and sJIA, which were positively correlated with the levels of serum ferritin. The authors assumed that the overproduction of IFN-γ, IL-18, and TNF-α could highlight the initiation of MAS, and that the serum concentrations of sTNFR-I for SLE, IL-18 for JDM, and sTNFR-II for KD and sJIA could be important biomarkers in the active phase of these diseases for the prediction of MAS onset [138].

The scientific studies discussed above to provide insight into the utility important cytokines as biomarkers are summarized in Table 3.

#### 2.3.8. CXCL9 Chemokine

Chemokines are a family of low-molecular-weight proteins that participate in inflammation as mediators, signaling molecules, migration stimulators and macrophage activators. There are four groups of ligands (XC, CC, CXC, and CX3C) for chemokines and over 20 rhodopsin-like receptors—7 of which are transmembrane—including CC receptors, CXC receptors, and receptors that exhibit non-G-protein-related signaling. 

Chemokines act on macrophage differentiation and polarization in physiological and pathological conditions, especially in inflammatory diseases. M1-type macrophages expose CXCL9 and CXCL10 chemokines, resulting from the activation of the cytokines by IFN-γ and TNFα, which attract Th1 lymphocytes. At the same time, the chemokines CXCL9 and CXCL10 work as attractants of macrophages in various inflammatory diseases (stomach burns, joints, etc.). It has been shown that osteoclast precursors exhibit the chemokine CXCR3, which can cross-react with the chemokine ligand CXCL9 and stimulate migration and differentiation of osteoclasts [136,139,140,141,142,143,144,145].

To define the cytokines and serum biomarkers involved in the onset of sJIA-MAS, Mizuta et al. used a special antibody array that simultaneously detects 174 cytokines to investigate samples from 15 sJIA patients, including 5 patients on TCZ therapy. Concentrations of five cytokines were highly increased in MAS compared to active sJIA. The chemokine CXCL9 showed the greatest increase after the onset of sJIA-MAS. Serum levels of CXCL9 were measured comparatively in 56 patients with sJIA, including 20 with MAS, to highlight the clinical value of CXCL9 as a biomarker of sJIA-MAS. The authors proved that CXCL9 was positively correlated with disease activity and that monitoring the serum levels of this chemokine could enable surveillance of sJIA-MAS [146].

Hinze et al. aimed to examine the associations of baseline serum biomarkers with the responses and outcomes of 54 active sJIA patients without recent MAS after canakinumab therapy. Most biomarkers were elevated at baseline in canakinumab-naïve patients compared to healthy controls, and some rapidly decreased by day 15 (i.e., IL-1Ra, IL-6, IL-18, and S100A12). Patients who responded to treatment had higher IL-18 and IFN-γ levels and lower CXCL9 levels at baseline, as documented by IL-18/CXCL9 and IFN-γ/CXCL9 ratios, which had significant accuracy in predicting treatment responses. The authors observed a differential regulation of the IL-18–IFN-γ–CXCL9 axis in patients with sJIA, and the higher the IL-18/CXCL9 and IFN-γ/CXCL9 ratios, the better the clinical response to canakinumab in sJIA [147].

#### 2.3.9. sCD163 and sCD25

In order to investigate MAS, which is a huge cascade of inflammatory phenomena triggered by the very intense activity of T cells and hemophagocytic macrophages, Bleesing et al. analyzed the concentrations of sIL2RA—also called sCD25 and soluble cluster of differentiation 163 (sCD163)—which reflected the levels of stimulation and expansion of these cells, for samples taken from 7 patients with sJIA-MAS compared with 16 patients with systemic sJIA at baseline without therapy, correlating these tests with serum ferritin levels and clinical symptoms. The mean level of sCD163 was 23,000 ng/mL in MAS, compared to only 5480 ng/mL in patients with active sJIA, while the mean level of sIL2RA was 19,646 pg/mL in MAS, compared to only 3787 pg/mL in active disease. In 5 out of 16 patients with sJIA, the serum concentrations of sIL2RA or sCD163 were similar in order of magnitude to those in MAS. It was concluded that sIL2RA and sCD163 could constitute sensitive biomarkers for the diagnosis of MAS, even in subclinical cases, and could also help identify patients with subclinical MAS [148].

Clinical and traditional laboratory markers of MAS, together with sCD25 and sCD163, were investigated by Reddy et al. in 33 patients with sJIA, 11 patients with active polyarticular JIA (polyJIA), and 2 patients with sJIA-MAS. Subjects with MAS had thrombocytopenia, leukopenia, and hypofibrinemia. The sCD25 value > 7500 pg/mL observed in MAS was also found in eight patients with active sJIA, who also had other highly altered laboratory values suggestive of MAS. Elevated levels of sCD63 (>1800 ng/mL) were detected in four patients with sJIA, and it was concluded that sCD25 > 7500 pg/mL could be a predictive biomarker of subclinical MAS [149].

Sakumura et al. conducted a study of 63 patients with sJIA, 4 with EBV-HLH, and 7 with KD, compared to 14 healthy controls, for whom the authors measured the serum concentrations of sCD163, neopterin, IL-18, and IL-6, and correlated them with the patients’ clinical symptoms. sCD163 was highly elevated in patients with sJIA-MAS and EBV-HLH compared to patients with only sJIA or KD in an acute phase. sCD163 increased dramatically with the evolution of MAS, being positively dependent on sJIA activity, even in patients under TCZ therapy. sCD163 showed a significant decrease in the inactive phase and later normalized, representing a potential diagnostic parameter for clinical remission in sJIA and an exclusive biomarker for the evaluation of disease activity, but also of MAS-sJIA [150].

Verweyen et al. conducted a study on gene expression in whole-blood samples taken from patients with sJIA at study entry and after 3 days of canakinumab therapy, compared to healthy controls. The authors found a distinct gene expression signature in patients with a very good clinical response to canakinumab compared to non-responders, mediated by upregulation of neutrophil-associated genes and IL-1, as well as an upregulated CD163 expression signature for the non-responders to biological therapy with canakinumab [151].

In a recent published review, Dik et al. concluded that in immune-mediated diseases where T-lymphocyte responses play an important role in the pathophysiological mechanisms, sCD25 could be a useful marker for prognosis or for monitoring the efficacy of immunosuppressive management [152].

#### 2.3.10. Neopterin

In a recent study, Takakura et al. comparatively investigated the serum concentrations of neopterin, IL-18, CXCL9, and sTNFR-I and II in 78 children diagnosed with sJIA, including 21 with MAS, to draw conclusions regarding the determination of the most accurate biomarkers for establishing the diagnosis of sJIA-MAS. From the performed analyses, the authors finally indicated neopterin as having the highest concentrations in sJIA-MAS and being positively correlated with transition to MAS from active sJIA; thus, neopterin could be a valuable future biomarker in sJIA-MAS [143].

#### 2.3.11. S100 Proteins

Various biomarkers are currently being researched for the diagnosis, monitoring of disease activity, predicting response to treatment, or the risk of recurrence in sJIA, AOSD, MAS, and other autoinflammatory diseases [57]. 

S100 proteins are a subgroup of the Ca^2+^-binding protein family; they are engaged in cell signal transduction, cell differentiation, motility regulation, transcription, and cell cycle progression, and extracellularly they perform a similar role to cytokines. The proteins S100A8/A9 (MRP8/14 or calprotectin) and S100A12 (calgranulin C) are secreted by phagocytic cells and may act as endogenous agonists of the toll-like receptor-4 and stimulate the innate immune response by releasing alarmins. These proteins have been identified as being more valuable than ESR and CRP as inflammatory biomarkers of the activity levels of rheumatic diseases. It is already known that the proteins S100A8/S100A9 and IL-1β play a special role in the pathogenesis of sJIA by activating phagocytic cells via two major signaling pathways of innate immunity [153,154,155].

S100 proteins, after being released by activated cells of the innate immune system (i.e., monocytes or neutrophils), play a special role in extending inflammation by binding to TLR4 and RAGE. TLR4 is known as a multiligand receptor for PAMPs of pathogenic bacteria, as well as DAMPs, while RAGE is a multiligand member of the cell surface molecule superfamily and interacts with various other molecules involved in homeostasis and inflammation. Both TLR4 and RAGE are expressed on the surface of immunological cells, endothelial cells, and other extravascular cells, so they participate in the onset and spread of systemic diseases during infection or inflammation [156,157,158].

Calprotectin, or CP—also known as MRP-8/MRP-14 or S100A8/A9 heterocomplex—released primarily by activated neutrophils, has been investigated in feces and used as a biomarker of inflammatory bowel disease and chronic rheumatic diseases in adults and children, including sJIA. CP functions through a mechanism termed “nutritional immunity”, by retaining transition metals essential for growth and vitality. Overaccumulation of CP could play a crucial role in inflammatory diseases, different types of arthritis, metastatic cancer, and septicemia. S100A8/A9 and S100A12 are valuable biomarkers for diagnosis, treatment response predictions, recurrence risk, and disease activity surveillance in sJIA [159,160,161,162].

Upon activation of neutrophils or monocyte endothelial adhesion, CP is secreted by an alternative microtubule-mediated, calcium-dependent pathway, along with protein kinase C (PKC), necrotic cells, and NETs, and may act as a marker for mononuclear phagocyte crowding at the site of inflammation [163].

Since CP is released at the site of inflammation, plasma CP concentrations have been proposed as a biomarker that represents the local activity of inflammatory disease, as opposed to CRP, which is produced mainly by hepatocytes during the nonspecific systemic inflammatory process. Being a relatively stable product, CP is more advantageous than IL-6, TNF-α, or IL-1β [164].

Altobelli et al. carried out a systematic review and meta-analysis of the role of CP as a potential biomarker of inflammation in JIA. The results showed a statistically significant difference between patients with the active systemic subtype compared to other subtypes of JIA, inactive disease, and healthy controls; the mean values of ESR and CRP were not found to be statistically significant. In conclusion, the evaluation of serum PC could provide us with data on the more accurate stratification of disease activity and the administration of more tailored and appropriate therapy in JIA [165].

Wittkowski et al. investigated the serum concentrations of the phagocytic pro-inflammatory protein S100A12 in serum samples taken from 240 patients with various pathologies with fever of unknown origin (FUO), diagnosed as follows: 60 with sJIA, 17 with familial Mediterranean fever (FMF), 18 with neonatal-onset multisystem inflammatory disease, 17 with Muckle–Wells syndrome, 40 with acute lymphoblastic leukemia, 5 with acute myeloblastic leukemia, and 83 with systemic infections, compared with 45 healthy controls. The samples were taken at the introduction to the study, before starting any kind of therapy. The results demonstrated a sensitivity of 66% and a specificity of 94% for S100A12 in differentiating sJIA from infections. The authors concluded that S100A12, as a biomarker of granulocyte activation, is highly overexpressed in patients with sJIA or FMF and can be a very valuable parameter in the evaluation and differential diagnosis of FUO, helping the doctor to make the correct decision to treat the patient with antibiotics or immunosuppressive agents [166].

Holzinger et al. studied the serum concentrations of myeloid-related protein complex 8 and 14 (MRP8/14) in 52 patients with sJIA to monitor disease activity and attempt a classification of patients at risk of relapse. The results indicated highly elevated serum concentrations of MRP8/14 in active disease and significantly reduced concentrations in the case of positive-response therapies. MRP8/14 faithfully reflected the clinical activity of sJIA, along with its inactivity or relapses, representing a valuable parameter for monitoring the response to treatment, being the first predictive biomarker for the subclinical activity of the disease, and allowing the stratification of patients at risk of relapse during periods of clinical inactivity of sJIA [167].

Starting from the hypothesis that the expression of programmed death ligand-1 (PD-L1)—an important immunoregulator (i.e., a glycoprotein expressed on antigen-presenting cells to limit T-lymphocyte activation during an inflammatory response)—is deregulated in sJIA monocytes, Shenoi et al. analyzed it in a pilot study on 20 patients (10 with active sJIA and 10 febrile non-sJIA). The authors compared PD-L1 expression in myeloid cells with other possible predictive biomarkers of sJIA, such as: CRP, ESR, leukocyte counts, S100A12, S100A8, S100A9, calprotectin, and procalcitonin, each used as a predictor independent of the other parameters in the analysis. The results highlighted that PD-L1 was significantly lower in sJIA, while S100 proteins were significantly increased with >80% sensitivity and >90% specificity in sJIA. The other investigated biomarkers were not found to be specific to sJIA, and the exploratory gene analysis indicated 106 genes as being important for sJIA, several of which were associated with immune response pathways. Although performed in a small cohort, S100 proteins stood out as specific diagnostic biomarkers for febrile patients with sJIA, and the decreased PD-L1 expression on the surface of circulating myeloid cells in sJIA could indicate a mechanism of loss of regulation of peripheral immunity [168].

Aljaberi et al. retrospectively investigated the usefulness of S100 protein testing in sJIA and other autoinflammatory and fever syndromes, in a pediatric clinical setting, to monitor the activity of each disease and support the diagnosis in 136 patients with the following pathologies: sJIA, non-systemic JIA (nsJIA), other defined autoinflammatory diseases (AIDs), and systemic undifferentiated recurring fever syndromes (SURFSs). In the case of sJIA, the concentrations of S100A8/9 and S100A12 were higher in active disease compared to inactive disease and had much higher values compared to all other categories included in the research; by comparison with CRP, they proved to be clearly superior in differentiating sJIA from AID and SURFS, and they were not associated with disease activity in nsJIA, AIDs, or SURFSs. The authors concluded that the significantly higher S100A8/9 and S100A12 protein levels in sJIA can support the correct diagnosis in this pathology, compared to other autoinflammatory and febrile diseases, and are superior to CRP in establishing the positive diagnosis of sJIA compared to other diagnoses, as well as when monitoring sJIA activity [169].

Because the differential diagnosis of sJIA from other infectious pathologies is sometimes very difficult in cases of prolonged fever in children, Park et al. investigated which would be the most useful biomarkers in diagnosis and tried to determine the usefulness of myeloid protein 8/14 (MRP8/14) measurements in order to transfer them from the laboratory to the bedside in clinical practice, in order to develop new methods of diagnosis for sJIA patients. Laboratory data from 1110 children and adolescents were divided into two cohorts: group A, to validate the performance of the MRP8/14 test by experimental ELISA, commercial ELISA, and an innovative lateral flow immunoassay (LFIA); and group B, to validate the accuracy of the diagnosis only with the last two tests. The authors found that MRP8/14 was increased in group A (*n* = 940) in sJIA compared to other conditions—including infections and autoinflammatory diseases—regardless of fever or the anti-inflammatory treatment received by the patient. In children and adolescents with fever, but without treatment (*n* = 195), serum concentrations of MRP8/14 in sJIA (19740 ± 5080 ng/mL) were much higher compared to other diagnoses, having the highest precision compared to all other parameters measured—such as ferritin, IL-18, ESR, soluble IL-2 receptor, and procalcitonin. The authors highlighted that serum tests of MRP8/14 can be useful for the positive diagnosis of sJIA in febrile patients, and commercial ELISA and LFIA allow accurate testing for rapid diagnostic screening at the point of care [18].

The lack of highly accurate biomarkers for monitoring disease activity—and as valuable tools for classifying and prognosticating treatment response or risk of flares in JIA patients—led La et al. to investigate serum calprotectin (sCP) concentrations in a multicenter cohort of 81 JIA patients, compared with 11 healthy controls. As compared to the controls, the researchers found that sCP values increased 8-fold in active sJIA and 2-fold in inactive disease, with greater specificity than CRP values and even stronger than ESH, allowing the differentiation of forms such as oligoarthritis, polyarthritis, and systemic forms, and recommending the future applications of sCP as a biomarker not only in the diagnosis and follow-up of JIA, but also for predicting relapses after stopping the treatment with non-steroidal anti-inflammatory drugs, methotrexate, or etanercept; thus, sCP should be implemented as soon as possible in clinical settings [170].

#### 2.3.12. Procalcitonin

First identified in the 1970s [171] as a precursor of calcitonin (a hormone involved in calcium homeostasis), procalcitonin (PCT) is a 116-amino-acid peptide generated by the parafollicular cells of the thyroid and the neuroendocrine cells of the intestine and the lungs. Plasma concentrations in healthy subjects are below the detection limit (0.01 µg/L) of clinical tests [172], increasing in response to pro-inflammatory stimuli—especially in bacterial infections—and considered to be an acute-phase reactant. In pediatric pathologies with high fever of unknown origin, an elevated plasma PCT level at the threshold of 0.5 ng/mL has a sensitivity and specificity of over 80% for the diagnosis of sepsis in infants and children [173].

Ninety-two subjects aged between 6 months and 18 years with untreated active JIA, inactive JIA, and healthy elective preoperative patients were included in a cohort study by Trachtman et al. to test the hypothesis that serum procalcitonin (sPCT) levels would differ between active JIA, inactive JIA, and healthy controls, by measuring their serum PCT and other common laboratory parameters of inflammation. The results indicated increased values for ESR, CRP, and PCT in patients with bacteremia compared to the other groups, with high rates of overlap between groups for ESR and CRP. The PCT concentration was equal to or greater than 0.15 μg/mL in only 1 out of 59 patients with JIA, whereas it was equal to or less than 0.15 μg/mL in only 1 patient with bacteremia. The authors’ conclusions were that although ESR, CRP, and PCT may all be biomarkers of choice for distinguishing JIA at presentation from serious bacterial infection (SBI), the most suitable biomarker appeared to be PCT, which was the most accurate in distinguishing between patients with infection and those with non-infectious inflammatory arthritis, which can greatly help clinicians to initiate the proper management immediately [174].

#### 2.3.13. sRAGE

Receptor for advanced glycation end products (RAGE; UniProtKB-Q15109) is a multiligand transmembrane receptor that is part of the immunoglobulin gene superfamily; 35 kD soluble cleaved RAGE—or soluble receptor for advanced glycation end products (sRAGE)—can be quantified in plasma or serum, and it has been found that high levels are associated with an increased risk of chronic diseases such as diabetes, atherosclerosis, coronary heart disease, chronic lung disease, etc. Increased sRAGE is positively associated with other pro-inflammatory advanced glycation end products (AGEs), and negatively with leukocytosis and increased levels of CRP and Fg [175,176,177,178].

Geroldi et al. presented an overview of the state of the art in the use of sRAGE as a biomarker and discussed its therapeutic potential for inflammatory diseases such as Alzheimer’s disease, atherosclerosis, and arthritis [179].

Elevated plasma levels of sRAGE may account for the potential anti-inflammatory and atheroprotective properties of this biomarker. sRAGE levels are inversely correlated with white blood cell counts and high-sensitivity C-reactive protein (hsCRP), and could indicate a slower rate of progression and a better prognosis of carotid artery disease [180].

In a recently published study, Prasad claimed that a low serum percentage of sRAGE cannot be considered a universal biomarker for patients with different pathologies compared to healthy control subjects. However, evidence supports the validity of a high AGE/sRAGE ratio as a universal biomarker/risk marker for diseases [181].

In an experimental murine model of cardiac ischemia/reperfusion (I/R), Zhang et al. demonstrated that sRAGE protected the heart from I/R lesions that could be induced by infiltration and proliferation of M1-type macrophages, which release IFN-γ following activation of the NF-κB signaling pathway by sRAGE in the differentiated M1 macrophages. The recruitment and proliferation of macrophages increased significantly in the presence of sRAGE [182].

#### 2.3.14. HMGB1

The high-mobility group box-1 (HMGB1; also called HMG1 or amphoterin) protein is the most plentiful member of the HMG family of DNA-binding proteins—a prototype of alarmins or DAMPs that are released into the extracellular space by activated or necrotic cells in viral and bacterial infections, as well as in several diseases with acute or chronic inflammation, such as cancer and ADs, and that work as key molecules connecting tissue damage and stress with innate immunity. In addition to its pro-inflammatory functions, HMGB1 also has effects in repairing and regenerating tissues. HMGB1 could be a therapeutic target in inflammatory chronic diseases. Recently generated HMGB1-specific inhibitors have been proposed as an interesting therapeutic target in inflammatory conditions that currently lack efficient therapies [183,184,185].

Together with extrinsic factors, intrinsic components can trigger an unpredictable immune response in sJIA. HMGB1 takes part in the pathogenesis of inflammatory diseases through interactions with pivotal transmembrane receptors, including RAGE and toll-like receptor-4 (TLR4). Macrophages and monocytes that are highly activated, along with disrupted TLR signaling and its undertrained ligands, are implicated in sJIA and AOSD. Extracellular HMGB1 has the ability to amplify inflammation and modulate immune responses in conjunction with RAGE. HMGB1 is an extracellular DAMP that binds to RAGE and TLR4 and spreads inflammatory signals. HMGB1 is involved in systemic inflammation from septicemic infections, arthritis, SLE, and liver damage [186,187,188,189].

The role of extracellular HMGB1 was discovered in 1999 as an important secreted protein in inflammation and infection. Extracellular HMGB1 transporting nucleic acids binds to RAGE and is internalized via endocytosis, so that the transferred nucleic acids interact with intracellular receptors and generate interferon and cytokine responses. HMGB1, as a pro-inflammatory mediator, binds several pattern-recognition receptors (PRRs); in addition to RAGE, members of the Toll-like receptor (TLR) family—i.e., TLR2, TLR4, and TLR9—as well as the CXC chemokine receptor 4 (CXCR4) and T-cell immunoglobulin mucin-3 (TIM-3) are released during apoptosis or necrosis, acting as DAMPs, while the inflammatory cytokines (i.e., TNF-α and IFN-γ) improve their release. When the concentration of the HMGB1 ligand increases, transmembrane RAGE is overexpressed in monocytes and macrophages and triggers an inflammatory immune response. sRAGE was shown to decrease the pro-inflammatory effects of HMGB1 [63,190,191,192].

Extracellular HMGB1 levels are increased in the inflamed joints of JIA patients and could be a biomarker of inflammatory activity and a target for therapy [193].

Bobek et al. investigated HMGB1 and sRAGE in a total of 144 enrolled children (97 with different subtypes of JIA, including sJIA; 19 with juvenile SLE; and 28 healthy controls), collecting consecutive samples of peripheral blood as well as synovial fluid (SF) at the time of diagnosis—prior to the initiation of treatment—and studying them in correlation with other common acute-phase reactants and clinical markers. The obtained results indicated much higher serum concentrations of HMGB1 and lower concentrations of sRAGE for children with JIA and those with SLE, respectively, compared to healthy controls, with positive correlations between HMGB1 and ESR, CRP, and α2 globulin, while the concentrations of sRAGE were inversely proportional to the same inflammatory markers in children with JIA. The median serum HMGB1 concentration in sJIA (17,402 pg/mL) was 15-fold higher than that in the control group (1149 pg/mL) and was also much higher than those in oligoarticular (3552 pg/mL), polyarticular (4374 pg/mL), and SLE patients (8523 pg/mL). Serum concentrations of HMGB1 were correlated with hepatosplenomegaly or serositis in sJIA. All JIA subgroups, as well as the SLE group, were characterized by lower serum levels of sRAGE, but the difference was significant only in patients with sJIA and in those with SLE. The inverse relationship between HMGB1 and its receptor sRAGE in blood and SF reflected that the inflammatory processes triggered by alarmins could play an important role in both investigated pathogeneses, and that HMGB1 could be an inflammatory biomarker to track in these patients as a potential target in the framework of biological therapy and for monitoring the activity of the disease [194].

Xu et al. conducted a prospective longitudinal study of 64 children with a mean age of 9.25 years and a mean duration of illness of 2.38 months, of whom 48.4% were female, and collected blood samples at the first visit and after 1 month, 3 months, and 6 months, for comparison to samples from healthy controls as well as from patients with reactive arthritis at the first visit. HMGB1 levels, routine laboratory data, and clinical disease characteristics were analyzed according to the protocol. The results at the first visit indicated significantly increased serum concentrations of HMGB1 in patients with sJIA compared to the other groups, as well as in enthesitis-associated arthritis compared to healthy controls. They also highlighted important correlations at the first visit between HMGB1 levels and disease duration, C-reactive protein, neutrophil percentage, and ferritin, suggesting that serum HMGB1 could be a sensitive inflammatory biomarker in sJIA. The authors recommended that further large-scale studies would be useful to explore the full range of HMGB1-related properties in JIA [195].

#### 2.3.15. Key Laboratory Data in sJIA-MAS

In order to confront the concentrations of CRP and ESR in children with JIA, and to trace their influence on diagnostic variables and prognostic factors, Wu et al. investigated 107 patients, of whom 18 had sJIA. At the time of diagnosis, the prevalence of ESR was much higher than that of CRP (86.8% vs. 47.2%) and was positively correlated with good response to therapy. On the other hand, the high concentrations of CRP at the beginning of the treatment were correlated with an inadequate response to the therapy and generally proved to be a predictive parameter of the failure of the first remission. This study demonstrated that ESR is useful in the diagnosis of sJIA—much more valuable than CRP—whereas a high initial CRP value may predict failure of remission [196].

Bloom et al. investigated 31 children with sJIA who were at high risk of developing severe disease based on their first measured D-dimer concentration. Ten children who initially had their risk category classified as “high” developed a severe outcome. The authors proved the paradigm that the risk of severe disease indicated by persistently elevated levels of fibrin D-dimer could be a prognostic parameter indicating a poor longer-term outcome in sJIA [197].

Follistatin-like protein 1 (FSTL-1) is known as a glycoprotein that is overexpressed in certain inflammatory diseases. Gorelik et al. investigated FSTL-1 concentrations in 28 patients with sJIA, including 7 patients who developed MAS, and compared them to 30 healthy controls, in correlation with already known biomarkers for the evaluation of sJIA activity. FSTL-1 gene expression concentrations were investigated in peripheral blood mononuclear cells (PBMCs) in correlation with ESR, ferritin, and sIL2RA. Serum concentrations of FSTL-1 were high in patients with sJIA at the beginning of the study, and they returned to normal over the following 24 months. The same high concentrations of FSTL-1 were also found in patients with acute MAS, which normalized after treatment. FSTL-1 was correlated with several serum markers of inflammation, including sIL2RA and ferritin. The ferritin/ESR ratio was superior to ferritin, sIL2RA, and FSTL-1 in differentiating MAS from new-onset sJIA. High serum concentrations of FSTL-1 detected before sJIA therapy are accompanied by dysregulated gene expression that may predict the presence of hidden MAS and progression to observable MAS. It has been shown that the serum ferritin/ESR ratio may be more valuable than ferritin alone in differentiating MAS from new-onset sJIA [198].

Minoia et al. conducted extensive research within a multinational collaborative project on 766 patients, of whom 362 had sJIA-MAS and 404 had active sJIA without MAS, in an attempt to develop and validate a score for the diagnosis of sJIA-MAS. The score for sJIA-MAS, named MS score, comprised seven variables: central nervous system (CNS) dysfunction, hemorrhagic manifestations, active arthritis, platelet count, Fg, LDH, and ferritin. The cutoff value ≥ −2.1 was the most reliable in differentiating MAS from active sJIA, with a sensitivity of 0.85 and a specificity of 0.95. The MS score could be a valuable practical instrument to be used by clinicians in the early diagnosis of sJIA-MAS [199].

Eloseily et al. investigated the value of the serum ferritin/ESR ratio for the diagnosis of sJIA-MAS and ferritin alone as a screening parameter for detecting MAS of other febrile etiologies. For sJIA patients with MAS (*n* = 362), without MAS (*n* = 404), and subjects (*n* = 345) hospitalized with systemic infection (SI), the ferritin/ESR ratio and ferritin alone were investigated to detect MAS among sJIA patients.

Meanwhile, the same parameters were investigated on a smaller number of cases with MAS of various causes, but also in patients hospitalized for fever. The ferritin/ESR ratio of 21.5 was 82% sensitive and 78% specific for the diagnosis of sJIA-MAS versus active sJIA without MAS. Only ferritin as a single laboratory parameter had a sensitivity of 95% (screening tool) and a specificity of 89.3% for differentiating patients with MAS from subjects with other febrile conditions. It was concluded that the serum ferritin/ESR ratio is a valuable parameter for the diagnosis of sJIA-MAS, and that the serum ferritin level alone can be used in screening to detect MAS among febrile patients [200].

In a multicenter study of 80 patients with MAS, Zou et al. included 53 cases of sJIA-MAS, 10 cases of KD-MAS, and 17 cases of connective tissue disease (CTD-MAS) and investigated the clinical symptoms and laboratory data before (pre-), at the onset, and during the complete phase of MAS. Among all patients, 51.2% presented MAS for the first time at the diagnosis of the underlying disease. Among patients with sJIA, 22.6% had hypotension before the onset of sJIA-MAS; in these patients, an increase in aspartate aminotransferase (AST) and lactate dehydrogenase (LDH) was observed, along with a decrease in serum albumin. Serum ferritin levels and ferritin/ESR ratios were greatly increased in subjects with active sJIA-MAS compared to the pre-MAS period. At the same time, a very high serum ferritin concentration and a significantly increased ferritin/ESR ratio were found in sJIA subjects compared to KD and CTD patients. In conclusion, almost half of the cases of MAS occurred when sJIA, KD, and CTD were present at first diagnosis. Hypotension was present in almost 30% of cases of MAS at onset, which could constitute a predictive aspect of the diagnosis. Increased serum ferritin, ferritin/ESR ratio, AST, and LDH, along with decreased serum albumin, could be predictive elements of the emergence of sJIA-MAS [201].

Ganeva et al. investigated biomarkers and their relationships with disease progression in the first year after diagnosis in a cohort of 266 JIA patients. Clinical symptoms and laboratory data were recorded quarterly in the first year and semiannually thereafter. The investigated biomarkers were increased in most JIA patients compared to healthy controls. In 10 subjects with sJIA, high concentrations of CRP, ESR, IL-18, S100A8/A9, and S100A12 were recorded compared to subjects with other JIA subtypes. Higher levels of ESR, G-CSF, IL-6, IL-17A, and TNF at baseline were predictive of a high risk of uninterrupted disease still active after 12 months. Elevated serum levels of CRP, S100A8/A9, and S100A12, as well as high ESR at baseline, were correlated with the necessity of intensified management with biological DMARDs in the first year of follow-up [202].

#### 2.3.16. Serum Amyloid A

Serum amyloid A is an acute-phase protein belonging to the group of apolipoproteins, synthesized by macrophages, adipocytes, synoviocytes, and especially hepatocytes. During the acute inflammatory process, it can increase significantly and has a chemotactic capacity for neutrophils and mast cells. SAA participates in the release of TNF-α, IL-1, IL-6, and granulocyte colony-stimulating factor (G-CSF); it stimulates angiogenesis, matrix metalloproteinases, and tissue factors. It is validated as a biomarker of the activity of diseases such as RA, JIA, rheumatic polymyalgia, ankylosing spondylitis, and sarcoidosis [203,204,205].

The main role of SAA in the pathogenesis of rheumatic inflammatory diseases is recognized by contemporary studies demonstrating its involvement in activating the inflammasome cascade and attracting IL-17-producing T-helper lymphocytes. From the earliest findings, it was clear that SAA could be used to monitor and assess the severity of disease activity in patients with rheumatoid arthritis and secondary amyloidosis. SAA has been observed to be a valuable biomarker in the immune pathology of rheumatic diseases, especially in the case of subclinical inflammation. Unlike other biomarkers, recent research has recognized the benefits of SAA monitoring in biological immunotherapy, especially after the implementation of proteomic techniques. Modern techniques encourage the research of SAA and its isoforms as sensitive biomarkers in rheumatic diseases—and even the development of therapeutic agents with SAA as a target. Another argument for recognizing its potential is given by recent findings that have shown that SAA is a very useful biomarker in monitoring severe coronavirus disease [206,207,208].

The clinical utility of SAA in both predicting and monitoring the response to therapy in patients with JIA has been less explored in recent decades. Dev et al. conducted a study on 50 newly diagnosed cases of JIA of all subtypes, along with 40 healthy controls, to find out whether SAA could be a biomarker of disease activity in JIA. The results showed that SAA concentrations were significantly higher in patients with JIA compared to healthy controls. Elevated SAA levels were positively correlated with disease activity in JIA, but not with CRP or ESR levels. The study showed that SAA is a more sensitive laboratory marker than ESR and CRP for assessing the presence of active joints [209].

#### 2.3.17. Leucine-Rich α2-Glycoprotein

Leucine-rich α2-glycoprotein (LRG) is a plasma glycoprotein whose physiological role is being studied. Recent data have highlighted the role of LRG in the differentiation and proliferation of Th17 lymphocytes and in the process of neovascularization by acting on the TGF-β in endothelial cells [210,211].

LRG is synthesized by the hepatocytes, and its concentration increases during inflammation under the action of the pro-inflammatory cytokines IL-1β, IL-6, and TNF-α, making it useful in diagnosing a multitude of inflammatory diseases (e.g., intestinal, rheumatoid arthritis, AOSD, sJIA, etc.). As part of the family of acute-phase proteins, similar to CRP and SAA, proteomic studies have shown that LRG is a sensitive biomarker in patients with rheumatoid arthritis, which increases during disease activity and can be reduced after anti-TNF-α therapy [212,213,214].

Leucine-rich α-2 glycoprotein 1 (LRG1) is an important member of the leucine-rich repetitive sequence protein family and is implicated in many human conditions, including cancer, diabetes, cardiovascular disease, neurological disease, inflammatory disorders, etc. It has recently been proven that LRG1 is compacted into the granules of human neutrophils and secreted upon the neutrophils’ stimulation to adjust the micro-medium. Serum LRG1 has been recognized as a practical biomarker for monitoring disease activity in patients with rheumatoid arthritis, lupus nephritis, adult-onset Still’s disease, and vasculitis. LRG1 is a recently detected important upstream signaling molecule of TGF-β that influences multiple pathophysiological mechanisms through the TGF-β signaling pathway [215,216,217].

In an early study, Shimizu et al. investigated the attributes of LRG as a biomarker for controlling sJIA activity during IL-6-targeting therapy. Serum LRG concentrations were investigated in four JIA patients treated with TCZ and correlated with clinical parameters and pro-inflammatory cytokines, including IL-18, IL-6, neopterin, and TNF-α receptor types I and II. Serum LRG concentrations increased simultaneously with the progression of active sJIA and the onset of MAS. Even when the disease was no longer active, serum LRG concentrations remained higher than normal values. A significant positive reciprocity was observed between serum levels of LRG and pro-inflammatory cytokines. Serum concentrations of LRG could probably be used as a unique biomarker for monitoring the progress of sJIA activity during IL-6 blocking therapy [218].

In another study, Shimizu et al. explored serum levels of LRG, IL-6, IL-18, sTNFR-I, and sTNFR-II in 59 patients with sJIA, 15 with other subtypes of JIA, 7 with KD, 7 with influenza A infection, 7 with infection with enterohemorrhagic *Escherichia coli* (EHEC), and 20 healthy controls (HCs) to whom the clinical symptoms were also compared. The serum LRG concentrations in active sJIA were much higher compared to other subtypes of JIA, EHEC, influenza patients, and HCs. Serum LRG values were normalized in patients with inactive sJIA after treatment and were positively correlated with those of serum CRP and serum ferritin. The authors claimed that serum LRG concentrations reflected the level of sJIA activity and could be useful as a biomarker for monitoring sJIA disease activity [219].

#### 2.3.18. Adenosine Deaminase 2

Lee et al. evaluated the serum levels of adenosine deaminase 2 (ADA2)—a protein with as-yet unknown function released mainly by monocytes and macrophages—as a novel biomarker of MAS in 324 healthy children and adults, and compared these serum levels with those of 173 children with inflammatory and immune-mediated diseases, including sJIA and nsJIA, KD, SLE, and JDM. The results showed increased levels of ADA2 in the peripheral blood of patients with sJIA and MAS; the activity of this protein was strongly correlated with the levels of ferritin, IL-18, and CXCL9 inducible by IFN-γ, but inconsistent with inflammatory markers (i.e., CRP and ESR). IL-18 and IFN-γ generated increased production of ADA2 by peripheral blood mononuclear cells, and ADA2 was abundant in MAS hemophagocytes. In conclusion, the authors noted the usefulness of research on serum ADA2 levels as a future biomarker for the diagnosis of sJIA-MAS [220].

The chemokines and other biomarkers discussed above are highlighted in Table 4.

## 3. Final Remarks and Conclusions

The COVID-19 pandemic—along with the associated CS—was and still is a challenge for 21st century medicine. Concretely, in the current crisis, solutions were sought, and initially an approach was even tried using some drugs inspired by rheumatology, including pediatric rheumatology. The emergence of multisystem inflammatory syndrome in children (MIS-C) was another test for pediatricians, because children and adolescents exposed to the SARS-CoV-2 virus—who, even if they initially only had mild forms of the disease, or were only contacts of people who were infected— developed a fulminant progression of a disease very similar to MAS after about 2–4 weeks, which put their lives in danger [11,221].

Being increasingly common, hyperinflammatory syndromes in pediatrics—including sJIA-MAS—rely on new biological disease-modifying anti-rheumatic drugs (bDMARDs) targeting various cytokines involved in the pathophysiological mechanisms of the diseases, which pediatric rheumatologists have begun to administer successfully more and more often. A CS, or hypercytokinemia, is initially a physiological response in which the innate immune system triggers an exaggerated and extreme discharge of pro-inflammatory signaling molecules, which would normally represent the immune system’s response to infection or immunotherapy, but the unexpected huge amounts of cytokines can cause multiorgan failure and death. There is still no single universally accepted definition of CS, as it differs depending on the corresponding inflammatory response [222].

CSS is an umbrella term for a systemic inflammatory condition [223] that results in the killing of both healthy and diseased cells, manifested by fever, fatigue, headache, rash, and muscle and/or joint aches, and may advance to more serious symptoms such as hypotension, tachycardia, vasodilatation, edema, hypoxemia, seizures, and even coma. Prompt and correct diagnosis, as well as the appropriate emergency management through immunosuppressive drugs, can hinder the irreversible multiorgan failure and save the patient’s life.

In pediatric pathology, within CSS, MAS can appear relatively frequently in children with sJIA—considered a prototype for this major complication that can even threaten their lives, just like MIS-C after infection with the SAR-CoV-2 virus during the COVID-19 pandemic. The major difficulty for pediatric rheumatologists is to make the correct differential diagnosis between active sJIA and MAS that has recently started or is already in progress, because these two entities have common clinical characteristics such as high fever, skin eruptions, and other systemic signs of inflammation which, if not identified in time, will evolve into hepatosplenic dysfunction, pancytopenia, consumption coagulopathy, hyperferritinemia and, finally, multiorgan failure [16,223,224].

sJIA-MAS continues to be a major problem in pediatric rheumatology due to its morbidity, mortality, and lack of standardization for early and successful diagnosis.

To be as close as possible to reality and clinical requirements, new model biomarkers are needed for an individualized approach to ensure the accurate assessment and management of patients with sJIA. In the current clinical practice generalized to date, a certain number of specific biomarkers for JIA have already been adopted by expert guidelines and are currently applied, such as ESR, CRP, serum ferritin, RF, HLA-B27 molecule, ANA, etc., which can help clinicians to better discriminate between the different subtypes and anticipate the response to the applied therapy, as well as relapses, which ideally should indicate the most appropriate medication for that patient and the point at which the therapy can be safely stopped after remission [225].

Already, in many clinics around the world that have significant funding for research and patient care—but especially those with ultrahigh-performance equipment and special analysis kits—new biomarkers are used to diagnose sJIA-MAS as quickly and accurately as possible and to predict the response to the established emergency treatment in this aggravating, disastrous, and potentially deadly complication of sJIA, which may be misleading for pediatric clinicians because it is so hard to diagnose and manage.

According to clinical studies, more than one-third of patients with sJIA may suffer from a subclinical form of MAS which, if recognized in time, could be managed long before reaching fully developed sJIA-MAS.

In children with subclinical sJIA-MAS, some biomarkers that highlight the extent of T-cell activation and are a measure for the escalation of phagocytic macrophages—such as sCD25 (sIL2RA) and sCD163—should be investigated to support the recognition of MAS.

In 2007, Bleesing et al. showed that elevated serum concentrations of sCD25 and sCD163 may be predictive biomarkers of sJIA-MAS and could help detect patients with subclinical MAS [148].

A few years later, Reddy et al. indicated that the presence of sCD25 levels > 7500 pg/mL can be considered to be a useful biomarker in the detection of patients with subclinical MAS [149].

In 2018, Sakumura et al., studying 63 patients with sJIA, concluded that sCD163 is a potential biomarker for the evaluation of disease activity and remission, faithfully reflecting macrophage activation in sJIA—even for patients treated with TCZ; its levels increased dramatically with the progression of MAS in sJIA [150].

In 2021, Verweyen et al. studied how distinct gene expression signatures are able to characterize strong clinical responders versus non-responders to canakinumab therapy in sJIA patients, and indicated that a signature including upregulated CD163 expression was associated with the non-response to this treatment [151].

Two other important biomarkers are the chemokines CXCL9 and CXCL10, which play an important role in the local recruitment of CD8^+^ T cells to tissues. Some scientific research has shown high serum concentrations of IFN-γ and IFN-γ-induced cytokines such as IL-18BP, CXCL9, and CXCL10 in sJIA-MAS, compared to their concentrations in active sJIA, inactive disease, or healthy controls. Serum values of CXCL9 and CXCL10 became smaller with remission of sJIA-MAS. Thus, these two biomarkers highlight the tissue activity of the IFN-γ pathway, and their ability to recruit CD8^+^ T cells—i.e., CXCL9 and CXCL10—correlates very well with the laboratory parameters of MAS, providing a strongly evidenced reason to act for in the vent of an IFN-γ blockage in sJIA-MAS [16,21,128,129]. 

According to other recent studies, CXCL9 has been shown to display the most significant increase after the onset of sJIA-MAS, and monitoring its serum levels would allow physicians to conduct surveillance of sJIA-MAS.

Concerning the response to canakinumab in sJIA, a differential regulation of the IL-18–IFN-γ–CXCL9 axis has been proven in patients with sJIA, and the higher the IL-18/CXCL9 and IFN-γ/CXCL9 ratios, the better the clinical response to canakinumab treatment [146,147].

Neopterin is released by macrophages stimulated by IFN-γ and could be a useful biomarker of immune system activation that can indicate disease activity in sJIA-MAS, while also allowing the diagnosis of the transition to MAS in active sJIA [143].

Neopterin could be an accurate biomarker for the evaluation of underlying inflammatory processes and the flares of sJIA, together with traditional laboratory parameters for diagnosis, clinical follow-up, and response to therapy. Concentrations of neopterin, IFN-γ, CXCL9, CXCL10, sCD25, and IL-18 can be used as biomarkers to determine the onset of MAS in active sJIA—even in children who have been prescribed TCZ [67,130,137,138].

Serum concentrations of MRP8/14 have been confirmed as an important biomarker for the diagnosis of sJIA, ensuring early discrimination from other inflammatory conditions so that targeted therapy for the patient’s benefit can be started immediately when the “window of opportunity” is open, stopping a possible persistent chronic outcome which, until the discovery of bDMARDs, was inevitable in more than 50% of patients [17,18,58,153].

The role of NK cells in sJIA-MAS has been widely investigated, but is still difficult to fully understand due to the small size or insufficient number of studies and conflicting conclusions. However, for sJIA-MAS, no strong genetic or transcriptional impairments in NK cells’ killing pathways were detected. Functional deficits of NK cells have been identified in sJIA-MAS due to inflammation-induced NK cell exhaustion. The high constitutive levels of cytokines (e.g., IL-6 and IL-18) could trigger the cytokine-induced NK cell dysfunction that fails to stop the immune response; thus, continuous inflammatory processes are generated [226].

The dramatic decrease in the number of figurative elements of the blood, coagulation abnormalities, significant liver cytolysis, and the increase in LDH as well as serum ferritin are important laboratory data for the identification of patients with sJIA-MAS. A concentration above 3500 μg/L has 85% sensitivity and 97% predictability for sJIA-MAS, and extreme hyperferritinemia detected at the time of sJIA diagnosis may be a biomarker to foreshadow sJIA-MAS [227].

Serum ferritin is accepted as an acute-phase reactant along with CRP, ESR, Fg, etc. In sJIA, high levels of ferritin and low levels of fibrinogen are inceptive evidence of inevitable sJIA-MAS [228].

High levels of platelets, CRP, and serum ferritin are specific biomarkers for the diagnosis MAS. Elevated serum ferritin concentrations in sJIA patients were correlated with active disease during progression, even when the disease activity decreased. Its status as a biomarker of disease activity is well-known, but the serum ferritin/ESR ratio may be a more valuable biomarker than ferritin alone in differentiating MAS from new-onset sJIA [200,229,230].

In discriminating between sJIA with and without MAS, the importance of the ferritin/ESR ratio, as well as the early identification of significant changes in serum ferritin and neutrophil, leukocyte, and platelet counts, is essential in the early stages of MAS, as evidenced by other recent studies [231].

Because many proposed new biomarkers will be difficult to implement in current clinical use due to their high cost or difficulties in implementation, an ideal candidate could be chosen from among the conventional laboratory biomarkers used already for assessing the evolution of clinical symptoms, such as the ferritin/ESR ratio, neutrophil/lymphocyte ratio, etc.

A neutrophil/lymphocyte ratio greater than 5.23 predicts the need for early biological treatment in sJIA and MAS [232].

Although sJIA-MAS was classified by expert consensus in 2016, today, after 6 years, new research insights are essential.

Biomarkers in sJIA-MAS could be relevant to differentiate disease subtypes, to assess disease status and activity, and to predict responses to initial treatment. They can also be very valuable tools in the pediatric rheumatologist’s decision to stop medication in a patient who has entered clinical remission.

There is no updated consensus regarding the validation, standardization, and reproducibility of methods for the determination and implementation of the most predictive biomarkers for the diagnosis of sJIA-MAS, the progress of MAS, the emergency treatment to be applied, the follow-up of the response, and the change of treatment in the event of emergency to save the life of the patient.

It was suggested that the limitations imposed by each method of determining a single biomarker could be overcome by using a comprehensive picture of the pathological aspects regarding the evolution of MAS and the very rapid use of several clinical parameters and laboratory data to include many more biomarkers. 

A clinically useful biomarker, in addition to being correctly measured, must have diagnostic and prognostic value in order to correlate well with the disease extent. It must also be reasonably stable, present in an easily accessible sample, and its measurement should be cost-effective. As of more recently, a multiple factor analysis that allows for simultaneous examination of multiple parameters—classified according to their physiological meaning—could be applied. On the other hand, in sJIA-MAS, the choice of biomarkers to be considered in the global assessment score should dictate their clinical relevance in the examined patients.

Machine learning analysis was efficient in differentiating between JIA patients and healthy controls, with ~90% precision. Analysis of the immune profiles could reveal immunological disturbances in patients with different JIA subtypes, but was much more important in patients with sJIA and in those with active disease. Such results open new perspectives for large-scale immunophenotyping in longitudinal studies of JIA, which will be able to better distinguish between JIA patients and healthy subjects, while the addition of machine learning could foretell the response to treatment [233].

**Figure 1:**
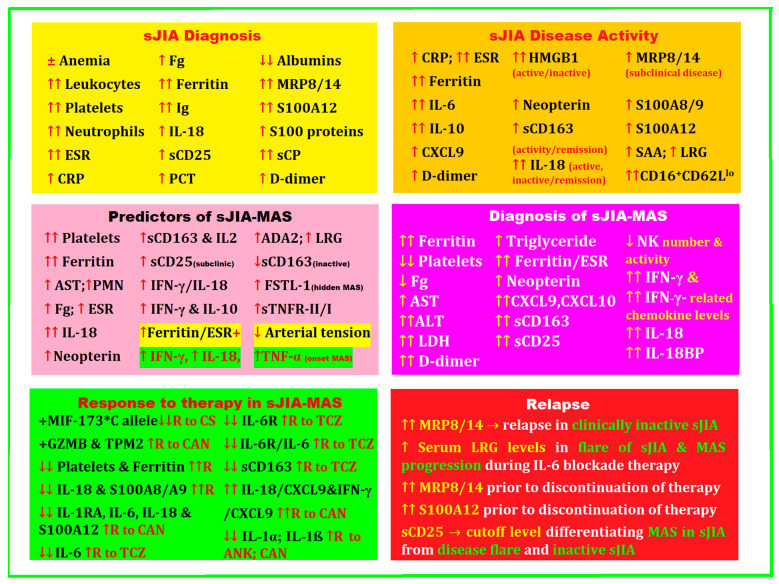
Biomarkers in sJIA and sJIA-MAS: diagnosis; disease activity; predictors of sJIA-MAS; diagnosis of sJIA-MAS; response to therapy in sJIA-MAS; and relapse. (Figure 1 was conceptualized and drawn by L.M.A. using Microsoft Paint 3D for Windows 10). Legend: [↑ = increasing/high; ↑↑ = very high; ↓ = decreasing/low; ↓↓ = very low; “+” = present; ± = present/absent; R = response].

The clinical significance of the biomarkers in sJIA and sJIA-MAS results from a comprehensive analysis of the biomarkers revealed in all of the abovementioned revised studies, and is highlighted as a synthesis in the following diagram (Figure 1):

Since the processes in sJIA-MAS take place extremely rapidly, it is imperative that the responsible pediatrician has adequate biomarkers to label the disease, initiate therapy, test correctly and quickly for response, and immediately prescribe a new drug if the patient does not respond.

sJIA and sJIA-MAS remain a call to participate in an open competition for clinicians to solve all of the questions that do not yet have a satisfactory answer, e.g., what are the most valuable biomarkers for an immediate and correct diagnosis? Which is the pathogenic pathway that needs to be blocked first? What is the best management practice? How should treatment be individualized and balanced?

The clinical significance of biomarkers in sJIA-MAS should be delineated by the latest scientific research, reanalyzed by a group of experts to draw new guidelines in clinical practice through an objective analysis of all of the relevant predictive biomarkers with great sensitivity and specificity, and to offer real support to clinicians to make the best decisions and choices under certain conditions—especially in the COVID-19 era.

## Figures and Tables

**Table 1:** Genetic biomarkers proposed for the diagnosis, activity, and prognosis of sJIA and MAS.

Reference/Year	Predictive Effects in sJIA and MAS
[20]. De Benedetti, F.; Meazza, C.; Vivarelli, M.; Rossi, F.; Pistorio, A.; Lamb, R.; Lunt, M.; Thomson, W.; Ravelli, A.; Donn, R.; et al. Functional and prognostic relevance of the −173 polymorphism of the macrophage migration inhibitory factor gene in systemic-onset juvenile idiopathic arthritis. *Arthritis Rheum*. **2003**, *48*, 1398–407. https://doi.org/10.1002/art.10882	Patients with sJIA onset with MIF-173*C-allele-associated persistent active disease have higher concentrations of MIF in serum and synovial fluid, as well as a weaker response to glucocorticoid therapy. The MIF-173*C-positive allele in patients with sJIA is a predictor of poor steroid therapy outcomes.
[21]. Yanagimachi, M.; Naruto, T.; Miyamae, T.; Hara, T.; Kikuchi, M.; Hara, R.; Imagawa, T.; Mori, M.; Sato, H.; Goto, H.; Yokota, S. Association of IRF5 polymorphisms with susceptibility to macrophage activation syndrome in patients with juvenile idiopathic arthritis. *J. Rheumatol.* **2011**, *38*, 769–774. https://doi.org/10.3899/jrheum.100655	*IRF5* gene polymorphism influences the susceptibility to MAS in sJIA, so IRF5 could trigger MAS in sJIA.
[22]. Canna, S.W.; de Jesus, A.A.; Gouni, S.; Brooks, S.R.; Marrero, B.; Liu, Y.; DiMattia, M.A.; Zaal, K.J.; Sanchez, G.A.; Kim, H.; et al. An activating NLRC4 inflammasome mutation causes autoinflammation with recurrent macrophage activation syndrome. *Nat. Genet.* **2014**, *46*, 1140–1146. https://doi.org/10.1038/ng.3089	An activating de novo mutation (c.1009A > T, p.Thr337Ser) in the nucleotide-binding domain of inflammasome component NLRC4 generates early-onset repeated episodes of fever, as well as MAS.
[23]. Ombrello, M.J.; Remmers, E.F.; Tachmazidou, I.; Grom, A.; Foell, D.; Haas, J.P.; Martini, A.; Gattorno, M.; Özen, S.; Prahalad, S. et al. International Childhood Arthritis Genetics (INCHARGE) Consortium. HLA-DRB1*11 and variants of the MHC class II locus are strong risk factors for systemic juvenile idiopathic arthritis. *Proc. Natl. Acad. Sci. USA* **2015**, *112*, 15970–15975. https://doi.org/10.1073/pnas.1520779112	HLA-DRB1*11 and its defining amino acid residue, glutamate 58, were strongly associated with sJIA, as well as the HLA-DRB1*11 haplotypes HLA-DQA1*05–HLA-DQB1*03.
[24]. De Silvestri, A.; Capittini, C.; Poddighe, D.; Marseglia, G.L.; Mascaretti, L.; Bevilacqua, E.; Scotti, V.; Rebuffi, C.; Pasi, A.; Martinetti, M.; et al. HLA-DRB1 alleles and juvenile idiopathic arthritis: Diagnostic clues emerging from a meta-analysis. *Autoimmun. Rev.* **2017**, *16*, 1230–1236. https://doi.org/10.1016/j.autrev.2017.10.007	HLA-DRB1*04 was confirmed to play a predisposing role in sJIA.
[25]. Arthur, V.L.; Shuldiner, E.; Remmers, E.F.; Hinks, A.; Grom, A.A.; Foell, D.; Martini, A.; Gattorno, M.; Özen, S.; Prahalad, S.; et al. IL1RN Variation Influences Both Disease Susceptibility and Response to Recombinant Human Interleukin-1 Receptor Antagonist Therapy in Systemic Juvenile Idiopathic Arthritis. *Arthritis Rheumatol.* **2018**, *70*, 1319–1330. https://doi.org/10.1002/art.40498	sJIA-associated SNPs were interdependent with *IL1RN* expression in LCLs, with an inverse correlation between sJIA risk and *IL1RN* expression. Homozygous high-expression alleles predicted the failure of anakinra therapy, making them ideal candidate biomarkers to manage the treatment of sJIA.
[26]. Hou, X.; Qu, H.; Zhang, S.; Qi, X.; Hakonarson, H.; Xia, Q.; Li, J. The Multi-Omics Architecture of Juvenile Idiopathic Arthritis. *Cells* **2020**, *9*, 2301. https://doi.org/10.3390/cells9102301	Several HLA alleles (both HLA class I and class II genes) and 23 non-HLA genetic loci were associated with different JIA subtypes. HLA class II alleles were significantly associated with sJIA. HLA-DRB1*11 had a strong association and SNP rs151043342 had the strongest association with sJIA. Nuclear factor interleukin-3-regulated gene (*NFIL3*) mutations drove elevated IL-1β, sensitizing patients to arthritis and rewiring the innate immune system for overproduction of IL-1. The laccase (multicopper oxidoreductase) domain-containing 1 (*LACC1*) gene regulated inflammation (i.e., macrophages, dendritic cells, and TNF-α levels). The Unc-13 homolog D (*UNC13D*) gene disrupted transcription factor binding.
[27]. Zhou, M.; Guo, R.; Wang, Y.F.; Yang, W.; Li, R.; Lu, L. Application of Weighted Gene Coexpression Network Analysis to Identify Key Modules and Hub Genes in Systemic Juvenile Idiopathic Arthritis. *BioMed Res. Int.* **2021**, *2021*, 957569. https://doi.org/10.1155/2021/9957569	The *ALAS2*, *AHSP*, TRIM10, *TRIM58*, and *KLF1* genes were associated with sJIA, but also with the differentiation of red blood cells, which may be related to anemia or MAS. The *KLRB1*, *KLRF1*, *CD160* and *KIR* genes might have a relationship with the dysregulation of NK cell function in sJIA. These results may deepen the comprehension of the pathophysiology of sJIA, and the hub genes may play the part of future biomarkers and treatment targets for sJIA.
[28]. Zhang, M.; Dai, R.; Zhao, Q.; Zhou, L.; An, Y.; Tang, X.; Zhao, X. Identification of Key Biomarkers and Immune Infiltration in Systemic Juvenile Idiopathic Arthritis by Integrated Bioinformatic Analysis. *Front. Mol. Biosci.* **2021**, *8*, 681526. https://doi.org/10.3389/fmolb.2021.681526	The hub genes *ARG1* and *PGLYRP1* are potential biomarkers for the early diagnosis of sJIA.
[29]. Ren, Y.; Labinsky, H.; Palmowski, A.; Bäcker, H.; Müller, M.; Kienzle, A. Altered molecular pathways and prognostic markers in active systemic juvenile idiopathic arthritis: integrated bioinformatic analysis. *Bosn. J. Basic Med. Sci.* **2022**, *22*, 247–260. https://doi.org/10.17305/bjbms.2021.6016	A total of 118 DEGs were identified, of which 94 were upregulated (most: *CD177*, *OLFM4*, *ARHGEF12*, *MMP8*, *PLOD2*, *CEACAM6*, and *CEACAM8*) and 24 were downregulated (most: *TCL1A*, *ALOX15*, and *HLA-DQB*) in active sJIA. A hub gene set of eight upregulated genes (*ARG1*, *DEFA4*, *HP*, *MMP8*, *MMP9*, *MPO*, *OLFM4*, and *PGLYRP1*) and none downregulated was also identified. *TPM2* and *GZMB* were identified as possible markers of positive short-term responses to canakinumab.

**Table 2:** Cellular biomarkers proposed for the diagnosis, activity, and prognosis of sJIA and MAS.

Reference/Year	Cellular Biomarkers	Predictive Effects in sJIA and MAS
[40]. Grom, A.A.; Villanueva, J.; Lee, S.; Goldmuntz, E.A.; Passo, M.H.; Filipovich, A. Natural killer cell dysfunction in patients with systemic-onset juvenile rheumatoid arthritis and macrophage activation syndrome. *J. Pediatr.* **2003**, *142*, 292–296. https://doi.org/10.1067/mpd.2003.110	**NK cells**	Cytotoxic activity and NK cell counts were decreased, and a slight increase in perforin expression in CD8^+^ and CD56^+^ cytotoxic cells was found, in most patients with sJIA-MAS. NK cell dysfunction is a frequent immunological abnormality for sJIA-MAS and fHLH.
[41]. Villanueva, J.; Lee, S.; Giannini, E.H.; Graham, T.B.; Passo, M.H.; Filipovich, A.; Grom, A.A. Natural killer cell dysfunction is a distinguishing feature of systemic onset juvenile rheumatoid arthritis and macrophage activation syndrome. *Arthritis Res. Ther.* **2005**, *7*, R30–R37. https://doi.org/10.1186/ar1453		NK cytolytic activity was much lower in sJIA patients than in other JIA groups or controls. The subgroup of JIA patients who had not yet experienced any MAS event had low NK function and a lack of circulating CD56^bright^ cells, similar to defects found in patients with MAS and HLH.
[42]. de Jager, W.; Vastert, S.J.; Beekman, J.M.; Wulffraat, N.M.; Kuis, W.; Coffer, P.J.; Prakken, B. Defective phosphorylation of interleukin-18 receptor beta causes impaired natural killer cell function in systemic-onset juvenile idiopathic arthritis. *Arthritis Rheum.* **2009**, *60*, 2782–2793. https://doi.org/10.1002/art.24750		NK cells from sJIA patients failed to phosphorylate the IL-18Rβ receptor following IL-18 stimulation, highlighting how NK cell dysfunction in sJIA is directly correlated with a defect in IL-18Rβ phosphorylation, which has important implications for understanding of the pathophysiological mechanisms and future therapy of sJIA.
[43]. Zhou, J.; Tang, X.; Ding, Y.; An, Y.; Zhao, X. Natural killer cell activity and frequency of killer cell immunoglobulin-like receptors in children with different forms of juvenile idiopathic arthritis. *Pediatr. Allergy Immunol.* **2013**, *24*, 691–696. https://doi.org/10.1111/pai.12130		In sJIA patients there was a decline in the activity of NK cells, which secreted more IFN-γ and less TNF-α, and the incidence of KIR2DS4 was reduced.
[44]. Put, K.; Vandenhaute, J.; Avau, A.; van Nieuwenhuijze, A.; Brisse, E.; Dierckx, T.; Rutgeerts, O.; Garcia-Perez, J.E.; Toelen, J.; Waer, M.; et al. Inflammatory Gene Expression Profile and Defective Interferon-γ and Granzyme K in Natural Killer Cells From Systemic Juvenile Idiopathic Arthritis Patients. *Arthritis Rheumatol.* **2017**, *69*, 213–224. https://doi.org/10.1002/art.39933		Increased IL-18 levels and a decreased IFN-γ/IL-18 ratio were found in sJIA patients. NK cells had an imbalance between inhibitory and activating receptors, with reduced G1 receptor expression and increased expression of NKp44. Hard-to-detect defects in the immune pathways governed by NK—such as the expression of granzyme K and the production of IFN-γ stimulated by IL-18—induced the immunoinflammatory dysfunctions that characterize sJIA.
[45]. Ohya, T.; Nishimura, K.; Murase, A.; Hattori, S.; Ohara, A.; Nozawa, T.; Hara, R.; Ito, S. Impaired Interleukin-18 Signaling in Natural Killer Cells From Patients With Systemic Juvenile Idiopathic Arthritis. *ACR Open Rheumatol.* **2022**, *4*, 503–510. https://doi.org/10.1002/acr2.11426		Increased levels of IL-18 induce the phosphorylation defects of MAPK and NF-κB in NK cells, and the inappropriate signaling of IL-18 in NK cells is directly related to the activity of sJIA.
[47]. Feng, D.; Huang, W.Y.; Niu, X.L.; Hao, S.; Zhang, L.N.; Hu, Y.J. Significance of Macrophage Subtypes in the Peripheral Blood of Children with Systemic Juvenile Idiopathic Arthritis. *Rheumatol. Ther.* **2021**, *8*, 1859–1870. https://doi.org/10.1007/s40744-021-00385-x	**Macrophages**	M1 induces inflammation in active sJIA, and M2a almost simultaneously triggers inflammation inhibition, while M2b and M2c play a major role in inhibiting inflammation in inactive sJIA. IL-6 had high levels in the group with active sJIA, while IL-4, IL-10, and IL-17 had high values in inactive disease.
[59]. Ter Haar, N.M.; Tak, T.; Mokry, M.; Scholman R.C.; Meerding, J.M.; de Jagerz, W.; Verwoerd, A.; Foell, D.; Vogl, T.; Roth, J. et al. Reversal of Sepsis-Like Features of Neutrophils by Interleukin-1 Blockade in Patients with Systemic-Onset Juvenile Idiopathic Arthritis. *Arthritis Rheumatol.* **2018**, *70*, 943–956. https://doi.org/10.1002/art.40442	**Neutrophils**	Neutrophils play an important role in sJIA, especially in the early inflammatory phase of the disease, and the neutrophil counts and the inflammatory activity in sJIA are both susceptible to IL-1 blockade.
[60]. Brown, R.A., Henderlight, M., Do, T., Yasin, S., Grom, A.A., DeLay, M., Thornton, S., Schulert, G.S. Neutrophils from Children with Systemic Juvenile Idiopathic Arthritis Exhibit Persistent Proinflammatory Activation Despite Long-Standing Clinically Inactive Disease. *Front. Immunol.* **2018**, *9*, 2995. https://doi.org/10.3389/fimmu.2018.02995		The results showed a higher percentage of the CD16^+^CD62L^lo^ neutrophil population in active sJIA than in the control group. Serum concentrations of S100 alarm proteins (i.e., S100A8/A9 and S100A12) were strongly correlated with the number of neutrophils in the peripheral blood, and analysis of the entire neutrophil transcriptome identified 214 differentially expressed (SD) genes compared to the neutrophils from controls. Neutrophil activation in patients with active sJIA or CID, together with the pro-inflammatory gene expression blueprint, demonstrated prolonged activation of innate immunity.
[64]. Kim, J.-W.; Ahn, M.-H.; Jung, J.-Y.; Suh, C.-H.; Kim, H.-A. An Update on the Pathogenic Role of Neutrophils in Systemic Juvenile Idiopathic Arthritis and Adult-Onset Still’s Disease. *Int. J. Mol. Sci.* **2021**, *22*, 13038. https://doi.org/10.3390/ijms222313038		Neutrophils, including NETs, play a pivotal role in the pathogenesis of sJIA and AOSD as future clinical biomarkers for monitoring and prognosis.
[65]. Ravelli, A.; Minoia, F.; Davì, S.; Horne, A.; Bovis, F.; Pistorio, A.; Aricò, M.; Avcin, T.; Behrens, E.M.; De Benedetti, F.; et al. Expert consensus on dynamics of laboratory tests for diagnosis of macrophage activation syndrome complicating systemic juvenile idiopathic arthritis. *RMD Open* **2016**, *2*, e000161. https://doi.org/10.1136/rmdopen-2015-000161	**Platelets**	Platelet counts, followed by ferritin levels, AST, white blood cell counts, neutrophil counts, fibrinogen, and ESR, were selected for the early diagnosis of MAS in sJIA.
[67]. De Matteis, A.; Pires Marafon, D.; Caiello, I.; Pardeo, M.; Marucci, G.; Sacco, E.; Prencipe, G.; De Benedetti, F.; Bracaglia, C. Traditional Laboratory Parameters and New Biomarkers in Macrophage Activation Syndrome and Secondary Hemophagocytic Lymphohistiocytosis [abstract]. *Arthritis Rheumatol.* **2020**, *72* (Suppl. S4). Available online: https://acrabstracts.org/abstract/traditional-laboratory-parameters-and-new-biomarkers-in-macrophage-activation-syndrome-and-secondary-hemophagocytic-lymphohistiocytosis/ (accessed on 21 August 2022).		Platelet count and ferritin are two relevant laboratory parameters, with high specificity and sensitivity, respectively, for the diagnosis of MAS in the context of sJIA.
[69]. Minoia, F.; Davì, S.; Horne, A.; Demirkaya, E.; Bovis, F.; Li, C.; Lehmberg, K.; Weitzman, S.; Insalaco, A.; Wouters, C.; et al. Clinical features, treatment, and outcome of macrophage activation syndrome complicating systemic juvenile idiopathic arthritis: a multinational, multicenter study of 362 patients. *Arthritis Rheumatol.* **2014**, *66*, 3160–3169. https://doi.org/10.1002/art.38802	**Complete blood cell count**	Typical laboratory features of sJIA include microcytic anemia; leukocytosis, thrombocytosis; elevated immunoglobulins; elevated ESR, CRP, and fibrinogen; and hypoalbuminemia.
[15]. Ravelli, A., Minoia, F.; Davì, S.; Horne, A.; Bovis, F.; Pistorio, A.; Aricò, M.; Avcin, T.; Behrens, E.M.; De Benedetti, F.; et al. 2016 Classification Criteria for Macrophage Activation Syndrome Complicating Systemic Juvenile Idiopathic Arthritis: A European League Against Rheumatism/American College of Rheumatology/Paediatric Rheumatology International Trials Organisation Collaborative Initiative. *Arthritis Rheumatol.* **2016**, *68*, 566–576. https://doi.org/10.1002/art.39332		MAS includes pancytopenia; increased levels of ferritin, liver enzymes (e.g., aspartate and alanine transaminases), triglycerides, and D-dimers; and hypofibrinogenemia.

**Table 3:** Important cytokines as biomarkers for the diagnosis, activity, and prognosis of sJIA and MAS.

Reference/Year	Cytokines as Biomarkers	Predictive Effects in sJIA and MAS
[9]. Ter Haar, N.M.; Van Dijkhuizen, E.H.P.; Swart, J.F.; Van Royen-Kerkhof, A.; El Idrissi, A.; Leek, A.P.; De Jager, W.; De Groot, M.C.H.; Haitjema, S.; Holzinger, D.; et al. Treatment to Target Using Recombinant Interleukin-1 Receptor Antagonist as First-Line Monotherapy in New-Onset Systemic Juvenile Idiopathic Arthritis: Results From a Five-Year Follow-Up Study. *Arthritis Rheumatol.* **2019**, *71*, 1163–1173. https://doi.org/10.1002/art.40865	**IL-1**	Treat-to-target management with rIL-1Ra as a first-line, short-term monotherapy may be a valuable option to interrupt the pathogenic cycle of sJIA, in which IL-1 is the cornerstone.
[80]. Saccomanno, B.; Tibaldi, J.; Minoia, F.; Bagnasco, F.; Pistorio, A.; Guariento, A.; Caorsi, R.; Consolaro, A.; Gattorno, M.; Ravelli, A. Predictors of Effectiveness of Anakinra in Systemic Juvenile Idiopathic Arthritis. *J. Rheumatol.* **2019**, *46*, 416–421. https://doi.org/10.3899/jrheum.180331		Univariate and multivariable statistical analyses of 62 patients diagnosed with sJIA, performed over a period of 14 years to measure the response to treatment with an IL-1 inhibitor (anakinra), identified the clinical profile of patients who can respond successfully to this therapy. Future in-depth studies are needed to detect the biomarkers that could accurately predict response to IL-1 or IL-6 antagonists.
[81]. Lainka, E.; Baehr, M.; Raszka, B.; Haas, J.P.; Hügle, B.; Fischer, N.; Foell, D.; Hinze, C.; Weissbarth-Riedel, E.; Kallinich, T.; et al. Experiences with IL-1 blockade in systemic juvenile idiopathic arthritis—Data from the German AID-registry. *Pediatr. Rheumatol. Online J.* **2021**, *19*, 38. https://doi.org/10.1186/s12969-021-00510-8		New strategies in the management of sJIA using IL-1 inhibitors—anakinra and canakinumab—were investigated in a cohort of patients, and the results demonstrated decreased disease activity, inflammatory parameters, and clinical symptoms.
[92]. Brunner, H.I.; Quartier, P.; Alexeeva, E.; Constantin, T.; Kone-Paut, I.; Marzan, K.; Schneider, R.; Wulffraat, N.M.; Chasnyk, V.; Tirosh, I.; et al. Efficacy and Safety of Canakinumab in Patients With Systemic Juvenile Idiopathic Arthritis With and Without Fever at Baseline: Results From an Open-Label, Active-Treatment Extension Study. *Arthritis Rheumatol.* **2020**, *72*, 2147–2158. https://doi.org/10.1002/art.41436	**IL-1β**	IL-1β, as part of the IL-1 family, plays a major role in autoinflammatory diseases, including sJIA. Inhibition of IL-1β rapidly blocks inflammation. Associated with systemic symptoms, IL-1β plays an important role in the pathogenesis of sJIA and the perpetuation of chronic inflammation. Canakinumab, as a monoclonal antibody that inhibits IL-1β and can decrease the levels of IL-6 and the hepatic synthesis of CRP and Fg, rapidly and persistently improved the clinical condition of patients with active sJIA, regardless of whether or not they had fever at the initiation of therapy.
[93]. Kostik, M.M.; Isupova, E.A.; Belozerov, K.; Likhacheva, T.S.; Suspitsin, E.N.; Raupov, R.; Masalova, V.V.; Chikova, I.A.; Dubko, M.F.; Kalashnikova, O.V.; et al. Standard and increased canakinumab dosing to quiet macrophage activation syndrome in children with systemic juvenile idiopathic arthritis. *Front. Pediatr.* **2022**, *10*, 894846. https://doi.org/10.3389/fped.2022.894846		An IL-1β monoclonal antibody (canakinumab), given at the onset of MAS-sJIA or even when MAS had developed during treatment with it, can be increased to 2–3 times the normal dose—albeit only in the short term—without any observed secondary effects, attesting to its efficacy in MAS-sJIA, but calling for further studies.
[95]. de Benedetti, F.; Massa, M.; Robbioni, P.; Ravelli, A.; Burgio, G.R.; Martini, A. Correlation of serum interleukin-6 levels with joint involvement and thrombocytosis in systemic juvenile rheumatoid arthritis. *Arthritis Rheum.* **1991**, *34*, 1158–1163. https://doi.org/10.1002/art.1780340912	**IL-6**	High serum IL-6 levels were correlated with the extent and severity of joint involvement, and these data suggest that this cytokine plays a significant role in the pathogenesis of sJIA.
[98]. Brachat, A.H.; Grom, A.A.; Wulffraat, N.; Brunner, H.I.; Quartier, P.; Brik, R.; McCann, L.; Ozdogan, H.; Rutkowska-Sak, L.; Schneider, R. et al. Pediatric Rheumatology International Trials Organization (PRINTO) and the Pediatric Rheumatology Collaborative Study Group (PRCSG). Early changes in gene expression and inflammatory proteins in systemic juvenile idiopathic arthritis patients on canakinumab therapy. *Arthritis Res. Ther.* **2017**, *19*, 13. https://doi.org/10.1186/s13075-016-1212-x		Analysis of IL-1β, IL-1 (IL1-R1 and IL1-R2), IL-1 receptor accessory protein (IL1-RAP), and IL-6 expression profiles in patients with sJIA after initiation of treatment with canakinumab found that the best clinical response was in patients with higher initial expression of disordered genes and a strong transcriptional response on day 3. Treatment interrupted a positive feedback loop between IL-1β signaling and IL-1β production. Canakinumab applied in sJIA patients resulted in downregulation of innate immune response genes and decreased IL-6 and clinical symptoms.
[103]. Shimizu, M.; Mizuta, M.; Okamoto, N.; Yasumi, T.; Iwata, N.; Umebayashi, H.; Okura, Y.; Kinjo, N.; Kubota, T.; Nakagishi, Y.; et al. Tocilizumab modifies clinical and laboratory features of macrophage activation syndrome complicating systemic juvenile idiopathic arthritis. *Pediatr. Rheumatol.* **2020**, *18*, 2. https://doi.org/10.1186/s12969-020-0399-1		TCZ, a humanized anti-IL-6 receptor monoclonal antibody, could modify the clinical and laboratory features of sJIA-MAS.
[104]. Qu, H.; Sundberg, E.; Aulin, C.; Neog, M.; Palmblad, K.; Horne, A.C.; Granath, F.; Ek, A.; Melén, E.; Olsson, M.; Harris, H.E. Immunoprofiling of active and inactive systemic juvenile idiopathic arthritis reveals distinct biomarkers: a single-center study. *Pediatr. Rheumatol.* **2021**, *19*, 173. https://doi.org/10.1186/s12969-021-00660-9		The biomarkers IL-6, IL-18, and S100A12 were confirmed to be increased during active sJIA as compared to healthy controls, and IL-18 was the only one with elevated levels in inactive sJIA. HMGB1 was found to be higher in active than in inactive sJIA. CASP8, CCL23, CD6, CXCL1, CXCL11, CXCL5, EIF4EBP1, KITLG, MMP1, OSM, SIRT2, SULT1A1, and TNFSF11 were found to be differentially expressed in active and/or inactive sJIA compared to the control group.
[107]. Imbrechts, M.; Avau, A.; Vandenhaute, J.; Malengier-Devlies, B.; Put, K.; Mitera, T.; Berghmans, N.; Burton, O.; Junius, S.; Liston, A. et al. Insufficient IL-10 Production as a Mechanism Underlying the Pathogenesis of Systemic Juvenile Idiopathic Arthritis. *J. Immunol.* **2018**, *201*, 2654–2663. https://doi.org/10.4049/jimmunol.1800468	**IL-10**	IL-10 was shown to be an immunosuppressive interleukin that counteracts IFN-γ activity, and disruption of normal IL-10 concentration led to the overproduction of IFN-γ, which participates in the pathogenesis of MAS. This study indicates that defective IL-10 production contributes to the pathogenesis of sJIA.
[108]. Peng, Y.; Liu, X.; Duan, Z.; Duan, J.; Zhou, Y. The Association of Serum IL-10 Levels with the Disease Activity in Systemic-Onset Juvenile Idiopathic Arthritis Patients. *Mediators Inflamm.* **2021**, *2021*, 6650928. https://doi.org/10.1155/2021/6650928		Patients with sJIA had higher serum concentrations of IL-10 compared to patients with other febrile illnesses. Serum IL-10 levels were much higher in active sJIA compared with inactive disease and were consistent with ESR, CRP, ferritin, and IL-6 levels. Serum IL-10 could be a valuable marker of sJIA activity.
[110]. Lotito, A.P.; Campa, A.; Silva, C.A.; Kiss, M.H.; Mello, S.B. Interleukin 18 as a marker of disease activity and severity in patients with juvenile idiopathic arthritis. *J. Rheumatol.* **2007**, *34*, 823–830.	**IL-18**	The levels of IL-18 and IL-6 in SF and serum were much higher in patients with sJIA than in other types of disease. The authors concluded that IL-18 is involved in the pathophysiology of JIA, reflects the severity of the disease, and could be a target for the treatment of arthritis.
[111]. Shimizu, M.; Yokoyama, T.; Yamada, K.; Kaneda, H.; Wada, H.; Wada, T.; Toma, T.; Ohta, K.; Kasahara, Y.; Yachie, A. Distinct cytokine profiles of systemic-onset juvenile idiopathic arthritis-associated macrophage activation syndrome with particular emphasis on the role of interleukin-18 in its pathogenesis. *Rheumatology* **2010**, *49*, 1645–1653. https://doi.org/10.1093/rheumatology/keq133		The serum level of IL-18 can be considered a biomarker of sJIA activity, and monitoring its profile could be beneficial for distinguishing MAS/HLH and estimating sJIA disease activity.
[112]. Shimizu, M.; Nakagishi, Y.; Inoue, N.; Mizuta, M.; Ko, G.; Saikawa, Y.; Kubota, T.; Yamasaki, Y.; Takei, S; Yachie, A. Interleukin-18 for predicting the development of macrophage activation syndrome in systemic juvenile idiopathic arthritis. *Clin. Immunol.* **2015**, *160*, 277–281. https://doi.org/10.1016/j.clim.2015.06.005		IL-18 could be involved in the pathophysiology of MAS, and serum concentrations > 47,750 pg/mL (cutoff value) could be useful in predicting the initiation of MAS.
[113]. Xia, Y.; Cui, P.; Li, Q.; Liang, F.; Li, C.; Yang, J. Extremely elevated IL-18 levels may help distinguish systemic-onset juvenile idiopathic arthritis from other febrile diseases. *Braz. J. Med. Biol. Res.* **2017**, *50*, e5958. https://doi.org/10.1590/1414-431X20165958		The levels of IL-18 in patients with sJIA were significantly higher than in other groups of febrile diseases. Serum IL-18 can be used as a biomarker to differentiate sJIA from other febrile diseases.
[115]. Kudela, H.; Drynda, S.; Lux, A.; Horneff, G.; Kekow, J. Comparative study of Interleukin-18 (IL-18) serum levels in adult-onset Still’s disease (AOSD) and systemic onset juvenile idiopathic arthritis (sJIA) and its use as a biomarker for diagnosis and evaluation of disease activity. *BMC Rheumatol.* **2019**, *3*, 4. https://doi.org/10.1186/s41927-019-0053-z		The results support the use of IL-18 as an important biomarker in AOSD and sJIA for disease activity.
[116]. Yasin, S.; Fall, N.; Brown, R.A.; Henderlight, M.; Canna, S.W.; Girard-Guyonvarc’hc, C; Gabay, C.; Grom, A.A.; Schulert, G.S. IL-18 as a biomarker linking systemic juvenile idiopathic arthritis and macrophage activation syndrome. *Rheumatology* **2020**, *59*, 361–366. https://doi.org/10.1093/rheumatology/kez282		Total IL-18 levels were significantly higher in patients with active sJIA and remained persistently elevated in the majority of those with inactive disease, where IL-18 could predict disease activity and history of MAS, respectively. In active disease, there was a moderate correlation between IL-18 and CXCL9, and a stronger correlation with serum ferritin.
[118]. Mizuta, M.; Shimizu, M.; Inoue, N.; Ikawa, Y.; Nakagishi, Y.; Yasuoka, R.; Iwata, N.; Yachie, A. Clinical significance of interleukin-18 for the diagnosis and prediction of disease course in systemic juvenile idiopathic arthritis. *Rheumatology* **2021**, *60*, 2421–2426. https://doi.org/10.1093/rheumatology/keaa634		IL-18 > 4800 pg/mL may be useful for the differentiation between sJIA and other diseases. Monitoring of serum IL-18 concentrations may be useful in predicting disease progression and assessing remission in sJIA.
[128]. Put, K.; Avau, A.; Brisse, E.; Mitera, T.; Put, S.; Proost, P.; Bader-Meunier, B.; Westhovens, R.; Van den Eynde, B.J.; Orabona, C.; et al. Cytokines in systemic juvenile idiopathic arthritis and haemophagocytic lymphohistiocytosis: tipping the balance between interleukin-18 and interferon-γ. *Rheumatology* **2015**, *54*, 1507–1517. https://doi.org/10.1093/rheumatology/keu524	**INF-γ**	Patients with active sJIA and HLH/MAS showed distinct cytokine profiles, with highly elevated plasma levels of IFN-γ and IFN-γ-induced proteins typically found in HLH/MAS. PBMCs, histiocytes, endothelial cells, and fibroblasts may contribute to an IFN-γ profile in plasma. Increasing levels of IFN-γ compared with IL-18 may raise suspicion about the development of MAS in sJIA.
[129]. Bracaglia, C.; de Graaf, K.; Pires Marafon, D.; Guilhot, F.; Ferlin, W.; Prencipe, G.; Caiello, I.; Davì.; S, Schulert, G.; Ravelli, A.; et al. Elevated circulating levels of interferon-γ and interferon-γ-induced chemokines characterise patients with macrophage activation syndrome complicating systemic juvenile idiopathic arthritis. *Ann. Rheum. Dis.* **2017**, *76*, 166–172. https://doi.org/10.1136/annrheumdis-2015-209020		Elevated levels of IFN-γ and IFN-γ-induced chemokines, in combination with other severely altered laboratory parameters in active MAS, support the significant involvement of IFN-γ in MAS.
[130]. Bracaglia, C.; Pires Marafon, D.; Caiello, I.; de Graaf, K.; Ballabio, M.; Ferlin, W.; Davì, S.; Schulert G.; Ravelli, A.; Grom, A.A.; et al. Biomarkers for the Diagnosis and the Identification of Risk of Macrophage Activation Syndrome (MAS) in Systemic Juvenile Idiopathic Arthritis (sJIA) [abstract]. *Arthritis Rheumatol.* **2017**, *69*. Available online: https://acrabstracts.org/abstract/biomarkers-for-the-diagnosis-and-the-identification-of-risk-of-macrophage-activation-syndrome-mas-in-systemic-juvenile-idiopathic-arthritis-sjia/ (accessed on 21 August 2022).		IFN-γ and IFN-γ-induced chemokines were significantly increased in active MAS and sec-HLH compared to active sJIA without MAS. In patients with active MAS, serum ferritin, alanine transferase, and neutrophil and platelet counts were significantly correlated with serum IFN-γ and CXCL9 levels. Significant involvement of IFN-γ in triggering MAS was observed.
[67]. De Matteis, A.; Pires Marafon, D.; Caiello, I.; Pardeo, M.; Marucci, G.; Sacco, E.; Prencipe, G.; De Benedetti, F.; Bracaglia, C. Traditional Laboratory Parameters and New Biomarkers in Macrophage Activation Syndrome and Secondary Hemophagocytic Lymphohistiocytosis [abstract]. *Arthritis Rheumatol.* **2020**, *72* (Suppl. S4). Available online: https://acrabstracts.org/abstract/traditional-laboratory-parameters-and-new-biomarkers-in-macrophage-activation-syndrome-and-secondary-hemophagocytic-lymphohistiocytosis/ (accessed on 21 August 2022).		Increased levels of IFN-γ-related biomarkers in patients with MAS and sec-HLH, together with platelet counts and ferritin levels, could be useful for diagnosis, clinical follow-up, and response to therapy.
[131]. Guo, L.; Xu, Y.; Qian, X.; Zou, L.; Zheng, R.; Teng, L.; Zheng, Q.; Leung Jung, L.K.; Lu, M. Sudden Hypotension and Increased Serum Interferon-γ and Interleukin-10 Predict Early Macrophage Activation Syndrome in Patients with Systemic Juvenile Idiopathic Arthritis. *J. Pediatr.* **2021**, *235*, 203–211.e3. https://doi.org/10.1016/j.jpeds.2021.02.008		Sudden onset of arterial hypotension, elevated ferritin/ESR ratio, and significantly high levels of IFN-γ and IL-10 are important markers for the early diagnosis of sJIA-MAS.
[135]. Hügle, B.; Hinze, C.; Lainka, E.; Fischer, N.; Haas, J.P. Development of positive antinuclear antibodies and rheumatoid factor in systemic juvenile idiopathic arthritis points toward an autoimmune phenotype later in the disease course. *Pediatr. Rheumatol. Online J.* **2014**, *12*, 28. https://doi.org/10.1186/1546-0096-12-28	**TNF-α**	Many patients developed positive ANA or positive RF, independent of the treatment with anti-TNF drugs, suggesting an autoimmune phenotype rather than an autoinflammatory one in the course of sJIA, probably involving the activation of B cells during the inflammatory process.
[136]. Shimizu, M.; Inoue, N.; Mizuta, M.; Nakagishi, Y.; Yachie, A. Characteristic elevation of soluble TNF receptor II:I ratio in macrophage activation syndrome with systemic juvenile idiopathic arthritis. *Clin. Exp. Immunol.* **2018**, *191*, 349–355. https://doi.org/10.1111/cei.13026		sTNFR-II/I had significantly higher levels in patients with sJIA-MAS. Serum IL-18 levels were elevated significantly in MAS patients. Higher serum IL-18 and sTNFR-II/I could be used as biomarkers for the diagnosis of sJIA-MAS, as well as for the differentiation between MAS and EBV-HLH.
[137]. Irabu, H.; Shimizu, M.; Kaneko, S.; et al. Comparison of serum biomarkers for the diagnosis of macrophage activation syndrome complicating systemic juvenile idiopathic arthritis during tocilizumab therapy. *Pediatr. Res.* **2020**, *88*, 934–939. https://doi.org/10.1038/s41390-020-0843-4		Serum sTNFR-II/I ratio could be a useful biomarker for assessing disease activity in sJIA-MAS, but also a predictive biomarker for the onset of MAS in the active phase of sJIA, even in patients under TCZ therapy.
[138]. Mizuta, M.; Shimizu, M.; Irabu, H.; Usami, M.; Inoue, N.; Nakagishi, Y.; Wada, T.; Yachie, A. Comparison of serum cytokine profiles in macrophage activation syndrome complicating different background rheumatic diseases in children. *Rheumatology* **2021**, *60*, 231–238. https://doi.org/10.1093/rheumatology/keaa299		Serum levels of sTNFR-I for SLE, IL-18 for JDM, and sTNFR-II for KD and sJIA could be useful diagnostic biomarkers for the transition from the active phase to MAS. Overproduction of IFN-γ, IL-18, and TNF-α might be closely related to the onset of MAS.

**Table 4:** Chemokines and other biomarkers for the diagnosis, activity, and prognosis of sJIA and MAS.

Reference/Year	Chemokines and Other Biomarkers	Predictive Effects in sJIA and MAS
[146]. Mizuta, M.; Shimizu, M.; Inoue, N.; Nakagishi, Y.; Yachie. A. Clinical Significance of Serum CXCL9 Levels as a Biomarker for Systemic Juvenile Idiopathic Arthritis Associated Macrophage Activation Syndrome. *Cytokine* **2019**, *119*, 182–187. https://doi.org/10.1016/j.cyto.2019.03.018	**CXCL9**	CXCL9 showed the most significant increase following the onset of sJIA-MAS and was positively correlated with disease activity. Monitoring the serum levels of this chemokine would enable surveillance of sJIA-MAS.
[147]. Hinze, T.; Kessel, C.; Hinze, C.H.; Seibert, J.; Gram, H.; Foell, D. A dysregulated interleukin-18-interferon-γ-CXCL9 axis impacts treatment response to canakinumab in systemic juvenile idiopathic arthritis. *Rheumatology* **2021**, *60*, 5165–5174. https://doi.org/10.1093/rheumatology/keab113		There was a differential regulation of the IL-18–IFN-γ–CXCL9 axis in patients with sJIA, and the higher the IL-18/CXCL9 and IFN-γ/CXCL9 ratios, the better the clinical outcome in response to canakinumab in sJIA.
[148]. Bleesing, J.; Prada, A.; Siegel, D.M.; Villanueva, J.; Olson, J.; Ilowite, N.T.; Brunner, H.I.; Griffin, T.; Graham, T.B.; Sherry, D.D.; et al. The diagnostic significance of soluble CD163 and soluble interleukin-2 receptor alpha-chain in macrophage activation syndrome and untreated new-onset systemic juvenile idiopathic arthritis. *Arthritis Rheum.* **2007**, *56*, 965–971. https://doi.org/10.1002/art.22416	**sCD163 and** **soluble IL-2Rα (sCD25)**	Elevated serum concentrations of sCD25 and sCD163 may be predictive biomarkers of MAS and help detect patients with subclinical MAS.
[149]. Reddy, V.V.; Myles, A.; Cheekatla, S.S.; Singh, S.; Aggarwal, A. Soluble CD25 in serum: a potential marker for subclinical macrophage activation syndrome in patients with active systemic onset juvenile idiopathic arthritis. *Int. J. Rheum. Dis.* **2014**, *17*, 261–267. https://doi.org/10.1111/1756-185X.12196		The presence of sCD25 levels > 7500 pg/mL can be considered a useful biomarker in the detection of patients with subclinical MAS.
[150]. Sakumura, N.; Shimizu, M.; Mizuta, M.; Inoue, N.; Nakagishi, Y.; Yachie, A. Soluble CD163, a unique biomarker to evaluate the disease activity, exhibits macrophage activation in systemic juvenile idiopathic arthritis. *Cytokine* **2018**, *110*, 459–465. https://doi.org/10.1016/j.cyto.2018.05.017		sCD163 might be a potential indicator of the disease activity and remission in sJIA and sJIA-MAS, as well as for patients receiving TCZ.
[151]. Verweyen, E.L.; Pickering, A.; Grom, A.A.; Schulert, G.S. Distinct Gene Expression Signatures Characterize Strong Clinical Responders Versus Nonresponders to Canakinumab in Children With Systemic Juvenile Idiopathic Arthritis. *Arthritis Rheumatol.* **2021**, *73*, 1334–1340. https://doi.org/10.1002/art.41640		A signature including upregulated CD163 expression was associated with non-response to canakinumab.
[143]. Takakura, M.; Shimizu, M.; Irabu, H.; Sakumura, N.; Inoue, N.; Mizuta, M.; Nakagishi, Y.; Yachie, A. Comparison of serum biomarkers for the diagnosis of macrophage activation syndrome complicating systemic juvenile idiopathic arthritis. *Clin. Immunol.* **2019**, *208*, 108252. https://doi.org/10.1016/j.clim.2019.108252	**Neopterin**	Serum neopterin levels may be used as an indicator of disease activity in sJIA and MAS, as well as for evaluating it. It may also be a useful marker to diagnose the transition to MAS from active sJIA.
[166]. Wittkowski, H.; Frosch, M.; Wulffraat, N.; Goldbach-Mansky, R.; Kallinich, T.; Kuemmerle-Deschner, J.; Frühwald, M.C.; Dassmann, S.; Pham, T.H.; Roth, J.; et al. S100A12 is a novel molecular marker differentiating systemic-onset juvenile idiopathic arthritis from other causes of fever of unknown origin. *Arthritis Rheum.* **2008**, *58*, 3924–3931. https://doi.org/10.1002/art.24137	**S100 proteins as follows:** **calgranulin A (S100A8) or MRP 8** **calgranulin B (S100A9/MRP14)** **calgranulin C (S100A12/MRP 6)** **calprotectin (CP) or MRP-8/MRP-14, or S100A8/A9 heterocomplex**	S100A12 is a useful biomarker in confirming the positive diagnosis of sJIA and excludes early severe systemic infections and several inflammatory or neoplastic disorders.
[167]. Holzinger, D.; Frosch, M.; Kastrup, A.; Prince, F.H.; Otten, M.H.; Van Suijlekom-Smit, L.W.; ten Cate, R.; Hoppenreijs, E.P.; Hansmann, S.; Moncrieffe, H.; et al. The Toll-like receptor 4 agonist MRP8/14 protein complex is a sensitive indicator for disease activity and predicts relapses in systemic-onset juvenile idiopathic arthritis. *Ann. Rheum. Dis.* **2012**, *71*, 974–980. https://doi.org/10.1136/annrheumdis-2011-200598		MRP8/14 (calprotectin) serum concentration was the first predictive biomarker indicating subclinical disease activity and stratifying patients at risk of relapse during clinically inactive sJIA.
[168]. Shenoi, S.; Ou, J.N.; Ni, C.; Macaubas, C.; Gersuk, V.H.; Wallace, C.A.; Mellins, E.D.; Stevens. A.M. Comparison of biomarkers for systemic juvenile idiopathic arthritis. *Pediatr. Res.* **2015**, *78*, 554–559. https://doi.org/10.1038/pr.2015.144		In a small cohort, S100 proteins were identified as specific diagnostic biomarkers for febrile patients with sJIA. Decreased PD-L1 surface expression on circulating myeloid cells in sJIA indicated a possible mechanism for the loss of peripheral immune regulation.
[169]. Aljaberi, N.; Tronconi, E.; Schulert, G.; Grom, A.A.; Lovell, D.J.; Huggins, J.L.; Henrickson, M.; Brunner, H. The use of S100 proteins testing in juvenile idiopathic arthritis and autoinflammatory diseases in a pediatric clinical setting: a retrospective analysis. *Pediatr. Rheumatol. Online J.* **2020**, *18*, 7. https://doi.org/10.1186/s12969-020-0398-2		S100A8/9 and S100A12 proteins were highly elevated in sJIA compared to nsJIA and other autoinflammatory diseases. Therefore, the authors concluded that S100 proteins (i.e., S100A8/9 and S100A12) are valuable biomarkers of disease activity in sJIA, but not in other autoinflammatory syndromes or nsJIA.
[18]. Park, C.; Miranda-Garcia, M.; Berendes, R.; Horneff, G.; Kuemmerle-Deschner, J.; Ganser, G.; Huppertz, H.I.; Minden, K.; Haas, J.P.; Jansson, A.F.; et al. MRP8/14 serum levels as diagnostic markers for systemic juvenile idiopathic arthritis in children with prolonged fever. *Rheumatology* **2021**, keab729. https://doi.org/10.1093/rheumatology/keab729		Compared with ferritin, IL-18, ESR, soluble IL-2 receptor, and procalcitonin, MRP8/14 showed the best accuracy in the diagnosis of sJIA.
[170]. La, C.; Lê, P.Q.; Ferster, A.; Goffin, L.; Spruyt, D.; Lauwerys, B.; Durez, P.; Boulanger, C.; Sokolova, T.; Rasschaert, J.; Badot, V. Serum calprotectin (S100A8/A9): a promising biomarker in diagnosis and follow-up in different subgroups of juvenile idiopathic arthritis. *RMD Open* **2021**, *7*, e001646. https://doi.org/10.1136/rmdopen-2021-001646		The study confirmed the potential uses of sCP as a biomarker in the diagnosis and follow-up of sJIA.
[174]. Trachtman, R.; Murray, E.; Wang, C.M.; Szymonifka, J.; Toussi, S.S.; Walters, H.; Nellis, M.E.; Onel, K.B.; Mandl, L.A. Procalcitonin Differs in Children With Infection and Children With Disease Flares in Juvenile Idiopathic Arthritis. *J. Clin. Rheumatol.* **2021**, *27*, 87–91. https://doi.org/10.1097/RHU.0000000000001170	**Procalcitonin**	The study found that CRP, ESR, and serum PCT levels are biomarkers that can be used to distinguish severe bacterial infection from active JIA at onset, but PCT was the most accurate. PCT can be used as a biomarker to help clinicians guide therapy.
[194]. Bobek, D.; Grčević, D.; Kovačić, N.; Lukić, I.K., Jelušić, M. The presence of high mobility group box-1 and soluble receptor for advanced glycation end-products in juvenile idiopathic arthritis and juvenile systemic lupus erythematosus. *Pediatr. Rheumatol. Online J.* **2014**, *12*, 50. https://doi.org/10.1186/1546-0096-12-50	**HMGB1/sRAGE** **AGEs and sRAGEs**	The study found positive correlations between serum HMGB1 and ESR, CRP, and α2 globulin—and vice versa for sRAGE—as inflammatory markers in children with sJIA. Elevated serum HMGB1 levels have been associated with hepatosplenomegaly and/or serosis in sJIA.
[195]. Xu, D.; Zhang, Y.; Zhang, Z.Y.; Tang, X.M. Association between high mobility group box 1 protein and juvenile idiopathic arthritis: a prospective longitudinal study. *Pediatr. Rheumatol.* **2021**, *19*, 112. https://doi.org/10.1186/s12969-021-00587-1		Serum HMGB1 could be considered to be a sensitive inflammatory biomarker for the assessment of clinical activity in JIA; it was found to be specifically elevated at first presentation in children with sJIA.
[196]. Wu, J.F.; Yang, Y.H.; Wang, L.C.; Lee, J.H.; Shen, E.Y.; Chiang, B.L. Comparative usefulness of C-reactive protein and erythrocyte sedimentation rate in juvenile rheumatoid arthritis. *Clin. Exp. Rheumatol.* **2007**, *25*, 782–785. PMID: 18078633	**Routine laboratory data in sJIA-MAS** **(CRP, ESR, Fg, D-dimer, serum ferritin, ferritin/ESR ratio, LDH, AST)**	ESR is a useful biomarker in the diagnosis of sJIA—much more valuable than CRP—whereas a high initial CRP value may strongly predict treatment failure.
[197]. Bloom, B.J.; Alario, A.J.; Miller, L.C. Persistent elevation of fibrin d-dimer predicts long term outcome in systemic juvenile idiopathic arthritis. *J. Rheumatol.* **2009**, *36*, 422–426. https://doi.org/10.3899/jrheum.070600		D-dimer could more accurately reflect the disease activity and prognosis as compared to clinical features in patients with sJIA.
[198]. Gorelik, M.; Fall, N.; Altaye, M.; Barnes, M.G.; Thompson, S.D.; Grom, A.A.; Hirsch, R. Follistatin-like protein 1 and the ferritin/erythrocyte sedimentation rate ratio are potential biomarkers for dysregulated gene expression and macrophage activation syndrome in systemic juvenile idiopathic arthritis. *J. Rheumatol.* **2013**, *40*, 1191–1199. https://doi.org/10.3899/jrheum.121131		In sJIA, elevated serum levels of FSTL-1 prior to treatment were related to a disorder of gene expression that predicts hidden MAS and progression to true MAS. Serum ferritin/ESR ratio may be superior to ferritin alone in discerning the obvious MAS from sJIA at onset.
[199]. Minoia, F.; Bovis, F.; Davì, S.; Horne, A.; Fischbach, M.; Frosch, M.; Huber, A.; Jelusic, M.; Sawhney, S.; McCurdy, D.K.; et al. Pediatric Rheumatology International Trials Organization, the Childhood Arthritis & Rheumatology Research Alliance, the Pediatric Rheumatology Collaborative Study Group and the Histiocyte Society. Development and initial validation of the MS score for diagnosis of macrophage activation syndrome in systemic juvenile idiopathic arthritis. *Ann. Rheum. Dis.* **2019**, *78*, 1357–1362. https://doi.org/10.1136/annrheumdis-2019-215211		MS score, which comprises seven variables (CNS dysfunction, hemorrhagic manifestations, active arthritis, platelet count, Fg, LDH, and ferritin), could be a valuable practical instrument that can be used by clinicians in the early diagnosis of sJIA-MAS.
[200]. Eloseily, E.M.; Minoia, F.; Crayne, C.B.; Beukelman, T.; Ravelli, A.; Cron, R.Q. Ferritin to erythrocyte sedimentation rate ratio: simple measure to identify macrophage activation syndrome in systemic juvenile idiopathic arthritis. *ACR Open Rheumatol.* **2019**, *1*, 345–349. https://doi.org/10.1002/acr2.11048		Serum ferritin/ESR ratio is a valuable parameter for the diagnosis of sJIA-MAS, and the serum ferritin level alone can be used in screening to detect MAS among febrile patients.
[201]. Zou, L.X.; Zhu, Y.; Sun, L.; Ma, H.H.; Yang, S.R.; Zeng, H.S.; Xiao, J.H.; Yu, H.G.; Guo, L.; Xu, Y.P.; Lu, M.P. Clinical and laboratory features, treatment, and outcomes of macrophage activation syndrome in 80 children: a multi-center study in China. *World J. Pediatr.* **2020**, *16*, 89–98. https://doi.org/10.1007/s12519-019-00256-0		Increased serum ferritin, ferritin/ESR ratio, AST, and LDH, along with decreased serum albumin, could be predictive parameters of the emergence of sJIA-MAS.
[202]. Ganeva, M.; Fuehner, S.; Kessel, C.; Klotsche, J.; Niewerth, M.; Minden, K.; Foell, D.; Hinze, C.H.; Wittkowski, H. Trajectories of disease courses in the inception cohort of newly diagnosed patients with JIA (ICON-JIA): the potential of serum biomarkers at baseline. *Pediatr. Rheumatol. Online J.* **2021**, *19*, 64. https://doi.org/10.1186/s12969-021-00553-x		Elevated baseline levels of CRP, S100A8/A9, and S100A12, as well as increased ESR, were associated with the necessity to escalate therapy during the first 12 months.
[209]. Dev, S.; Singh, A. Study of role of serum amyloid A (SAA) as a marker of disease activity in juvenile idiopathic arthritis. *J. Fam. Med. Prim. Care* **2019**, *8*, 2129–2133. https://doi.org/10.4103/jfmpc.jfmpc_339_19	**SAA**	SAA is a more sensitive laboratory marker than ESR and CRP for assessing the presence of active joints in JIA.
[218]. Shimizu, M.; Nakagishi, Y.; Inoue, N.; Mizuta, M.; Yachie, A. Leucine-rich α2-glycoprotein as the acute-phase reactant to detect systemic juvenile idiopathic arthritis disease activity during anti-interleukin-6 blockade therapy: A case series. *Mod. Rheumatol.* **2017**, *27*, 833–837. https://doi.org/10.1080/14397595.2016.1270795	**LRG**	Serum LRG levels might be a potential biomarker of sJIA disease activity during IL-6 blockade treatment.
[219]. Shimizu, M.; Inoue, N.; Mizuta, M.; Nakagishi, Y; Yachie, A. Serum Leucine-Rich α2-Glycoprotein as a Biomarker for Monitoring Disease Activity in Patients with Systemic Juvenile Idiopathic Arthritis. *J. Immunol. Res.* **2019**, *2019*, 3140204. https://doi.org/10.1155/2019/3140204		Serum LRG levels were positively correlated with serum CRP and ferritin levels in sJIA and reflected disease activity; thus, they may be useful for monitoring sJIA disease activity.
[220]. Lee, P.Y.; Schulert, G.S.; Canna, S.W.; Huang, Y.; Sundel, J.; Li, Y.; Hoyt, K.J.; Blaustein, R.B.; Wactor, A.; Do, T.; et al. Adenosine deaminase 2 as a biomarker of macrophage activation syndrome in systemic juvenile idiopathic arthritis. *Ann. Rheum. Dis.* **2020**, *79*, 225–231. https://doi.org/10.1136/annrheumdis-2019-216030	**ADA2**	The utility of research on plasma ADA2 activity as a future diagnostic biomarker of sJIA-MAS was demonstrated. The monocyte/macrophage origin of ADA2 and induction by IL-18 and IFN-γ provide further support for the key role of MAS in this life-threatening complication of sJIA.

## Data Availability

Data supporting reported results can be found by the first author L.M.A.

## Abbreviations

ALL	Acute lymphoblastic leukemia
ADA2	Adenosine deaminase 2
AOSD	Adult-onset Still’s disease
AGEs	Advanced glycation end products
AHSP	Alpha hemoglobin-stabilizing protein
ACR	American College of Rheumatology
ACR 50	American College of Rheumatology score = at least 50% improvement in disease activity
ANK	Anakinra
ANAs	Antinuclear antibodies
Arg-1	Arginase-1
AST	Aspartate aminotransferase
ADs	Autoimmune diseases
AID	Autoinflammatory disease
bDMARDs	Biological disease-modifying anti-rheumatic drugs
S100A12	Calgranulin C
CP, or S100A8/A9, or MRP8/14	Calprotectin
CAN	Canakinumab
CASP8	Caspase-8
CCL23	C-C motif chemokine ligand 23
CDC	Centers for Disease Control and Prevention
CNS	Central nervous system
CXCL1…11	Chemokine (C-X-C motif) ligand 1…11
CCL2	Chemokine (C-C motif) ligand 2
CXCL9	Chemokine (C-X-C motif) ligand 9
CXCL10	Chemokine (C-X-C motif) ligand 10
CX3CR1	Chemokine (C-X3-C motif) receptor 1
CID	Clinically inactive disease
CD	Cluster of differentiation
CD163	Cluster of differentiation 163
CLPs	Common lymphoid progenitors
CBC	Complete blood count
CTD	Connective tissue disease
cNK	Conventional NK
csDMARDs	Conventional synthetic disease-modifying anti-rheumatic drugs
CS	Corticosteroid
CRP	C-reactive protein
CSS	Cytokine storm syndrome
DAMP	Damage-associated molecular pattern
DNA	Deoxyribonucleic acid
DEGs	Differentially expressed genes
DMARDs	Disease-modifying anti-rheumatic drugs
EHEC	Enterohemorrhagic *Escherichia coli*
ELISA	Enzyme-linked immunosorbent assay
EBV	Epstein–Barr virus
EBV-HLH	Epstein–Barr-virus-induced hemophagocytic lymphohistiocytosis
ESR	Erythrocyte sedimentation rate
fHLH	Familial hemophagocytic lymphohistiocytosis
FMF	Familial Mediterranean fever
FasL	Fas ligand
FUO	Fever of unknown origin
Fg	Fibrinogen
FSTL-1	Follistatin-like protein 1
FDA	Food and Drug Administration
CFA	Freund’s complete adjuvant
GEO	Gene Expression Omnibus
G-CSF	Granulocyte colony-stimulating factor
GM-CSF	Granulocyte–macrophage colony-stimulating factor
GZMK	Granzyme K
HCs	Healthy controls
HSC	Hematopoietic stem cell
HLH	Hemophagocytic lymphohistiocytosis
HMGB1	High-mobility group box-1
hsCRP	High-sensitivity C-reactive protein
HLA	Human leukocyte antigen
Igs	Immunoglobulins
ILCs	Innate lymphoid cells
ILC1	“Unconventional” NK cells
ICAM-5	Intercellular adhesion molecule 5
IFN-α	Interferon-alpha
IFN-γ	Interferon-gamma
IRF5	Interferon regulatory factor 5
IL-1, IL-6, IL-18	Interleukin 1, 6, 18
IL-18Rβ	IL-18β receptor
IL-1 alpha, IL-1α; IL-1 beta, IL-1β	Interleukin 1 alpha, beta
IL-1Ra	Interleukin-1 receptor antagonist
IL10RA	Interleukin-10 receptor, alpha subunit
IL6ST	Interleukin-6 signal transducer
IL-18BP	IL-18-binding protein
ICAM-1	Intracellular adhesion molecule 1
IV	Intravenous
JDM	Juvenile dermatomyositis
JIA	Juvenile idiopathic arthritis
JADAS	Juvenile Arthritis Disease Activity Score
KD	Kawasaki disease
LDH	Lactate dehydrogenase
LRG	Leucine-rich *α*2-glycoprotein
LRG1	Leucine-rich α-2 glycoprotein 1
LPSs	Lipopolysaccharides
CD62L	L-selectin
LCLs	Lymphoblastoid cell lines
M	Macrophage
M1	M1 macrophages (encourage inflammation)
M2	M2 macrophages (decrease inflammation and encourage tissue repair)
MAS	Macrophage activation syndrome
sJIA-MAS	Macrophage activation syndrome in sJIA
MIF	Macrophage migration inhibitory factor
MHC	Major histocompatibility complex
MMP1	Matrix metalloproteinase 1
MMP3	Matrix metalloproteinase 3
MEFV	Mediterranean fever
MTX	Methotrexate
MAPKs	Mitogen-activated protein kinases
MCP1	Monocyte chemoattractant protein 1
MIS-C	Multisystem inflammatory syndrome in children
MPO–DNA complexes	Myeloperoxidase–DNA complexes
MRP	Myeloid-related protein
MRP8	Myeloid-related protein 8
MRP14	Myeloid-related protein 14
NCR2 or NKp44	Natural cytotoxicity-triggering receptor 2
NK	Natural killer
NKT	Natural killer T
NCAM or CD56	Neural cell adhesion molecule, also called CD56
NETs	Neutrophil extracellular traps
NETosis	NET activation and release
NLR	NOD-like receptor
NSAIDs	Non-steroidal anti-inflammatory drugs
nsJIA	Non-systemic forms of JIA
NFIL3	Nuclear factor, interleukin 3-regulated gene
NF-κB or NF-kappaB	Nuclear factor kappa-light-chain-enhancer of activated B cells
NOD	Nucleotide-binding oligomerization
NLRP	Nucleotide-binding oligomerization domain, leucine-rich repeat and Pyrin domain
NLRP3	Nucleotide-binding oligomerization domain-like receptor pyrin domain-containing protein 3
NLRs	Nucleotide-binding oligomerization domain-like receptors = *NOD*-like receptors
No.	Number; Latin “numero”, the ablative case of “numerus”
PAMP	Pathogen-associated molecular pattern
PRRs	Pattern-recognition receptors
PRINTO	Pediatric Rheumatology International Trials Organization
PRF1	Perforin 1
PBMC	Peripheral blood mononuclear cell
PMLs or PMNLs	Polymorphonuclear leukocytes
PMN	Polymorphonuclear neutrophil
+	Present
±	Present/absent
pHLH	Primary hemophagocytic lymphohistiocytosis
PCT	Procalcitonin
PD-L1	Programmed death ligand-1
PKC	Protein kinase C
qRT-PCR	Quantitative reverse-transcription polymerase chain reaction
ROS	Reactive oxygen species
TNFSF11	Receptor activator of nuclear factor-κB ligand, RANKL
RAGE	Receptor for advanced glycation end products
rIL-1Ra	Recombinant IL-1 receptor antagonist
rr	Reference range
R	Response
RMD	Rheumatic and musculoskeletal disease
RF	Rheumatoid factor
S100A9	S100 calcium-binding protein A9
MS score	Score for sJIA-MAS
sec-HLH	Secondary hemophagocytic lymphohistiocytosis
SAA	Serum amyloid A
sCP	Serum calprotectin
sPCT	Serum procalcitonin
SIF	Severe infections
SNP	Single-nucleotide polymorphism
sCD163	Soluble cluster of differentiation 163
sCD25 or sIL2RA	Soluble IL-2 receptor alpha chain, or soluble IL2RA
sRAGE	Soluble receptor for advanced glycation end products
sTNFR-I	Soluble tumor necrosis factor receptor I
sTNFR-II	Soluble tumor necrosis factor receptor II
sTNF-R II/I	Soluble TNF receptor II/I
SF	Synovial fluid
sJIA	Systemic juvenile idiopathic arthritis
SLE	Systemic lupus erythematosus
TCR	T-cell receptor
Th cells, CD4+ or CD4-positive	T-helper cells, or CD4+ cells, or CD4-positive cells
trNK	Tissue-resident (tr)NK
TWEAK	TNF-like weak inducer of apoptosis
TCZ	Tocilizumab
TLR	Toll-like receptor
TGF-β	Transforming growth factor beta
TAMs	Tumor-associated macrophages
TNF-α	Tumor necrosis factor alpha
TNFR1	Tumor necrosis factor receptor 1
FcγRIII	Type III Fcγ receptor
WGCNA	Weighted gene co-expression network analysis
WT	Wild type
Increasing/high	↑
Very high	↑↑
Decreasing/low	↓
Very low	↓↓
